# Transcriptional Corepressors HIPK1 and HIPK2 Control Angiogenesis Via TGF-β–TAK1–Dependent Mechanism

**DOI:** 10.1371/journal.pbio.1001527

**Published:** 2013-04-02

**Authors:** Yulei Shang, Christina N. Doan, Thomas D. Arnold, Sebum Lee, Amy A. Tang, Louis F. Reichardt, Eric J. Huang

**Affiliations:** 1 Department of Pathology, University of California San Francisco, San Francisco, California, United States of America; 2 Pathology Service 113B, VA Medical Center, San Francisco, California, United States of America; 3 Department of Physiology, University of California San Francisco, San Francisco, California, United States of America; 4 Department of Pediatrics, University of California San Francisco, San Francisco, California, United States of America; Duke University Medical Center, United States of America

## Abstract

Several critical events dictate the successful establishment of nascent vasculature in yolk sac and in the developing embryos. These include aggregation of angioblasts to form the primitive vascular plexus, followed by the proliferation, differentiation, migration, and coalescence of endothelial cells. Although transforming growth factor–β (TGF-β) is known to regulate various aspects of vascular development, the signaling mechanism of TGF-β remains unclear. Here we show that homeodomain interacting protein kinases, HIPK1 and HIPK2, are transcriptional corepressors that regulate TGF-β–dependent angiogenesis during embryonic development. Loss of HIPK1 and HIPK2 leads to marked up-regulations of several potent angiogenic genes, including *Mmp10* and *Vegf*, which result in excessive endothelial proliferation and poor adherens junction formation. This robust phenotype can be recapitulated by siRNA knockdown of *Hipk1* and *Hipk2* in human umbilical vein endothelial cells, as well as in endothelial cell-specific TGF-β type II receptor (TβRII) conditional mutants. The effects of HIPK proteins are mediated through its interaction with MEF2C, and this interaction can be further enhanced by TGF-β in a TAK1-dependent manner. Remarkably, TGF-β-TAK1 signaling activates HIPK2 by phosphorylating a highly conserved tyrosine residue Y-361 within the kinase domain. Point mutation in this tyrosine completely eliminates the effect of HIPK2 as a transcriptional corepressor in luciferase assays. Our results reveal a previously unrecognized role of HIPK proteins in connecting TGF-β signaling pathway with the transcriptional programs critical for angiogenesis in early embryonic development.

## Abstract 2

During angiogenesis, endothelial cells in nascent blood vessels use the transcriptional cofactors HIPK1 and HIPK2 to transduce TGF-β signals and control cell proliferation and adhesion.

## Author Summary

An essential step during early embryonic development is to establish elaborate vascular networks that provide nutrients to ensure the proper growth of the embryos. This process, known as angiogenesis, requires coordinated regulation of cell proliferation, migration, and differentiation in endothelial cells, which provide the inner-most linings of blood vessels. It is well accepted that transforming growth factor–β (TGF-β) and its downstream signal pathways are required to regulate endothelial cell growth, but the exact mechanisms remain poorly characterized. Using mouse genetics and in vitro angiogenesis assays, we show that transcriptional cofactors in the homeodomain interacting protein kinase (HIPK) family are activated by TGF-β to control the expression of target genes that regulate proliferation and adherent junction formation in endothelial cells. Our study also identifies a highly conserved tyrosine residue in HIPK proteins that is required to transduce TGF-β signal. These results provide new insights into the mechanism of TGF-β signaling in angiogenesis, and how this process may be exploited to develop therapeutic targets that control angiogenesis during development and in disease conditions.

## Introduction

Vascular morphogenesis is controlled by an intricate interplay of extrinsic factors and their downstream signaling mechanisms [1],[2]. At the early stage of vascular development, several critical events dictate the successful establishment of nascent vasculature in yolk sac and in the developing embryos. These include aggregation of angioblasts to form the primitive vascular plexus, followed by the proliferation, differentiation, migration, and coalescence of endothelial cells [2],[3]. Subsequently, branching morphogenesis and arteriovenous specification further facilitate the maturation of an interconnecting and fully functional network of blood vessels to provide nutrients to the entire organism [4]. Many of the mechanisms that govern the normal vascular development can also be recapitulated in angiogenesis that occurs during disease conditions, including tumorigenesis, metastasis, stroke, and tissue repair after injury [1],[5].

Transforming growth factor–β (TGF-β) represents a family of highly conserved cytokines that have profound effects in regulating epithelial–mesenchymal transition (EMT), vascular morphogenesis, and cellular and organismal functions during development and in disease conditions [6]–[8]. Indeed, genetic analyses in mouse and human have shown that mutations involving components of the TGF-β signaling pathway affect many aspects of vascular morphogenesis during development and in adult life [9]. For instance, loss-of-function analyses of TGF-β1, TGF-β type I receptor ALK1 or ALK5, or TGF-β type II receptor (TβRII) in mouse reveal a distinct role of each of these signaling components in regulating the proliferation, differentiation, and survival of endothelial cells and smooth muscle cells. These analyses further indicate that the outcome of the deletion involving different components of the TGF-β signaling pathway can be cell context-dependent. Furthermore, the timing of targeted deletion and the presence of genetic modifiers can also affect the phenotypic manifestations [7]. With respect to the roles of TGF-β signaling in endothelial functions, TGF-β type I receptors ALK1 and ALK5 have been shown to have opposite effects, with ALK1 contributing to the proliferation and migration of endothelial cells and ALK5 inducing the maturation of blood vessels [10],[11]. While the underlying mechanisms for distinct effects of ALK1 and ALK5 are still unclear, it is possible that the signaling downstream of the TGF-β type I receptors may diverge due to the involvement of Smad and non-Smad-dependent mechanisms that regulate the transcription of angiogenesis-related genes [12].

Homeodomain interacting protein kinase 2 (HIPK2) is a transcriptional cofactor in the downstream of TGF-β/BMP signaling pathway [13]–[17]. Interestingly, loss of HIPK2 reduces cellular responses to TGF-β during neuronal development and in mouse models of renal fibrosis [13],[17]. While mice lacking HIPK1 show no detectable defects [18], simultaneous loss of HIPK1 and HIPK2 leads to severe growth retardation and early embryonic lethality [19],[20]. Although the study by Aikawa and colleagues has implicated vascular defects in *Hipk1* ^−*/*−^ *;Hipk2* ^−*/*−^ double mutants [20], the detailed mechanism responsible for the phenotypes remains unclear. It is also unclear if HIPK1 and HIPK2 can cooperatively regulate TGF-β signaling and thereby contribute to the angiogenesis during early embryonic development.

Here, we show that HIPK1 and HIPK2 cooperatively suppress the expression of angiogenic genes that are critical for endothelial proliferation and adherens junction formation. Loss of HIPK1 and HIPK2 leads to a marked up-regulation of VEGF and MMP10, and early embryonic lethality due to excessive proliferation and poor adherens junction formation in the endothelial cells. Consistent with these results, siRNA knockdown of *Hipk1* and *Hipk2* results in similar phenotype in human umbilical vein endothelial cells (HUVECs). Furthermore, endothelial cell-specific deletion of TβRII results in phenotypes similar to those in *Hipk1* ^−*/*−^ *;Hipk2* ^−*/*−^ mutants. The mechanism of HIPK1 and HIPK2 involves their interaction with HDAC7 to suppress MEF2C-mediated transcriptional activation of *Mmp10* and *Vegf*. Importantly, the activity of HIPK critically depends on the TGF-β-TAK1 mechanism, which promotes the phosphorylation of HIPK2 on a highly conserved tyrosine residue in the kinase domain. Together, these results provide novel insights into the role of HIPK1 and HIPK2 in the signal transduction mechanism downstream of TGF-β and the transcriptional control of angiogenic gene expression during the critical stages of vascular morphogenesis.

## Results

### Increased Proliferation and Poor Adherens Junction Formation in Endothelial Cells Lacking HIPK1 and HIPK2

**Figure 1:**
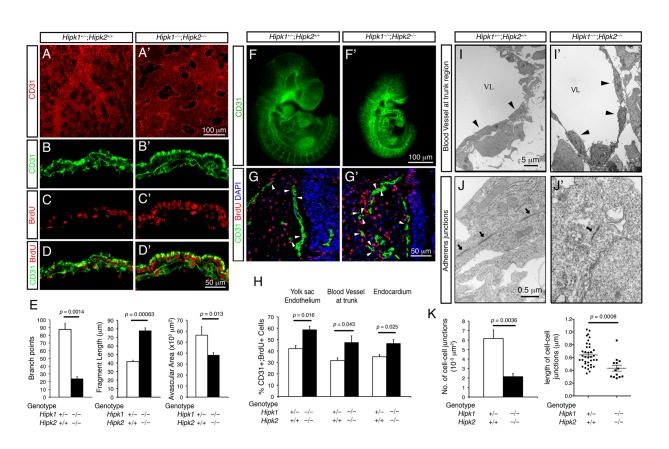
Increased proliferation and poor adherens junction formation in the endothelial cells of *Hipk1* ^−^ ^***/*−**^ ***;Hipk2*** **^−^** ^***/*−**^ **embryos.** (A–D) Whole-mount and confocal immunofluorescent images of the developing vasculature in the yolk sacs of *wild-type* and *Hipk1* ^−*/*−^ *;Hipk2* ^−*/*−^ embryos. (E) Quantification of the branch points, vessel lengths, and avascular areas in E9.5 yolk sacs. (F) Whole-mount CD31 staining of E9.5 *control* and *Hipk1* ^−*/*−^ *;Hipk2* ^−*/*−^ embryos. (G) Transverse sections of E9.5 embryos at the trunk level. Arrowheads in panels (G) and (G') indicate the blood vessels in control and *Hipk1* ^−*/*−^ *;Hipk2* ^−*/*−^ mutant embryos. (H) Quantification of CD31+;BrdU+ endothelial cells in E9.5 yolk sacs, trunk blood vessels, or endocardium. (I–J') EM analyses show reduced size and density of adherens junctions in *Hipk1* ^−*/*−^ *;Hipk2* ^−*/*−^ endothelial cells. Panels (I) and (I') are low-magnification EM images, whereas (J) and (J') are high-magnification images. Arrowheads in (I) and (I') indicate intact endothelial cells that show no evidence of leakiness. Arrows in (J) and (J') highlight the presence of adherens junctions in both control and *Hipk1* ^−*/*−^ *;Hipk2* ^−*/*−^ mutants, though the size and density of adherens junction are reduced in *Hipk1* ^−*/*−^ *;Hipk2* ^−*/*−^ mutants. (K) Quantification shows the reduced length and density of adherens junction in E9.5 *Hipk1* ^−*/*−^ *;Hipk2* ^−*/*−^ mutants.

To determine if HIPK1 and HIPK2 cooperatively regulate gene expression, we analyzed vascular development in *Hipk1* ^−*/*−^ *;Hipk2* ^−*/*−^ mutants. In contrast to the previous report [20], CD31 (PECAM-1) staining in the yolk sacs of E9.5 *Hipk1* ^−*/*−^ *;Hipk2* ^−*/*−^ mutants showed an excessive growth of endothelial cells, with reduced avascular areas, reduced vascular branch points, increased fragment length, and a significant increase in BrdU incorporation (Figure 1A–E,H). Similar vascular phenotypes, including increase in endothelial cell proliferation and vascular density, were also detected in the endothelial cells in the head and trunk regions of E9.5 *Hipk1* ^−*/*−^ *;Hipk2* ^−*/*−^ (Figure 1F–H). Electron microscopy further revealed that the adherens junctions in the endothelial cells of *Hipk1* ^−*/*−^ *;Hipk2* ^−*/*−^ mutants were significantly smaller and showed reduced density per unit area compared to those in control (*Hipk1^+/^* ^−^ *;Hipk2^+/+^*) (Figure 1I–K). Despite these defects, the endothelial cells in *Hipk1* ^−*/*−^ *;Hipk2* ^−*/*−^ mutants showed no evidence of disruption or disorganization, and blood cells remained confined within the vessels with no evidence of vascular leakiness (Figure 1I–I'). Another prominent phenotype in *Hipk1* ^−*/*−^ *;Hipk2* ^−*/*−^ mutants was the absence of blood vessel growing into the neural tubes (Figure 1G–G'), which may have contributed to the increase in cell death and reduced proliferation in the neural progenitors in *Hipk1* ^−*/*−^ *;Hipk2* ^−*/*−^ mutants [19].

### Expression Profiling in *Hipk1* ^−*/*−^ *;Hipk2* ^−*/*−^ Embryos Revealed Abnormal Regulations in TGF-β Targets and Angiogenesis Genes

To investigate the molecular bases of the *Hipk1* ^−*/*−^ *;Hipk2* ^−*/*−^ mutant phenotype, we used the CodeLink Mouse Whole Genome Bioarrays to characterize gene expression profiles in E9.5 control (*Hipk1^+/^* ^−^ *;Hipk2^+/+^*), *Hipk1* ^−*/*−^ *;Hipk2^+/+^*, *Hipk1^+/^* ^−^ *;Hipk2* ^−*/*−^, and *Hipk1* ^−*/*−^ *;Hipk2* ^−*/*−^ embryos. Unsupervised hierarchical clustering analyses of all genes showed that the transcriptomes of *Hipk1* ^−*/*−^ *;Hipk2^+/+^* embryos were more similar to that of control (*Hipk1^+/^* ^−^ *;Hipk2^+/+^*), whereas the profiles of *Hipk1^+/^* ^−^ *;Hipk2* ^−*/*−^ were more similar to *Hipk1* ^−*/*−^ *;Hipk2* ^−*/*−^ embryos (Figure S1A). Consistent with this, Gene Ontogeny and KEGG pathway analyses indicated that only a very small number of genes in *Hipk1* ^−*/*−^ *;Hipk2^+/+^* embryos showed altered expression patterns. In contrast, the number of affected genes in each pathway showed a progressive increase from *Hipk1* ^−*/*−^ *;Hipk2^+/+^*, *Hipk1^+/^* ^−^ *;Hipk2* ^−*/*−^, to *Hipk1* ^−*/*−^ *;Hipk2* ^−*/*−^ mutants (Figure S1B). Together, these results supported the idea that HIPK1 and HIPK2 regulated target genes expression in a cooperative and interdependent manner.

**Figure 2:**
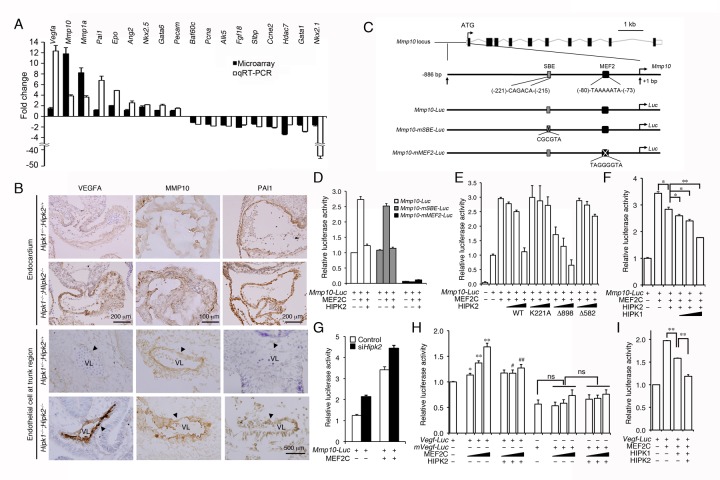
HIPK1 and HIPK2 suppress MEF2C-mediated *Mmp10* and *Vegf* expression. (A) Expression profiles of *Hipk1* ^−*/*−^;*Hipk2* ^−*/*−^ embryos reveal marked up-regulation of several angiogenic genes. The mRNA levels in *Hipk1* ^−*/*−^ *;Hipk2* ^−*/*−^ mutants are normalized to those in control. (B) Immunohistochemical analyses confirmed the increased expression of VEGFA, MMP10, and PAI1 in the endocardium and endothelial cells of *Hipk1* ^−*/*−^;*Hipk2* ^−*/*−^ embryos. Arrowheads indicate endothelial cells, and VL stands for vascular lumen. (C) Schematic diagrams of the 886-bp upstream regulatory sequence of the *Mmp10* locus. The potential binding sites for SBE and MEF2 and the mutations of SBE or MEF2 sites are shown. (D) MEF2 binding element, but not the SBE, is required for HIPK2 to suppress *Mmp10*-luciferase activity. (E) *Mmp10*-luciferase reporter assays in HEK293T cells show that the kinase activity and the protein–protein interacting domain of HIPK2 are required to suppress MEF2C-mediated activation of *Mmp10* expression. (F) HIPK1 and HIPK2 cooperatively suppress MEF2C-mediated activation of *Mmp10* reporter. (G) Acute knockdown of *Hipk2* in HEK293T cells using siRNA promotes the activation of *Mmp10*-luciferase reporter in the absence or presence of MEF2C. (H) *Vegf*-luciferase reporter can be activated by MEF2C and suppressed by HIPK2 in HEK293T cells. Student's *t* test, *n* = 3 (*p<0.05, **p<0.01, when compared to *Vegf-Luc* alone; ^#^ *p*<0.05, ^##^ *p*<0.01, when compared to the same condition without exogenous HIPK2). (I) HIPK1 and HIPK2 show cooperative and additive effects in suppressing MEF2C-mediated *Vegf-Luc* activity. Data are shown as mean ± s.e.m. Student's *t* test, *n* = 3 (*p<0.05, **p<0.01).

Given the role of HIPK2 in the TGF-β-BMP signaling pathways [13],[14], we next asked if the concomitant loss of HIPK1 and HIPK2 could affect the expression of TGF-β-BMP downstream targets. Consistent with this idea, a number of TGF-β target genes were either up- or down-regulated in *Hipk1* ^−*/*−^ *;Hipk2* ^−*/*−^ embryos (Table S1). These included genes related to vascular development (e.g., *Pai-1*) [21] or cell cycle regulation (e.g., *Cdkn2c*, *Cyclin E2*, *Pcna*) (Figure 2A and Figure S1C) [22]–[24]. Remarkably, further analyses of the HIPK1/2 targets revealed several additional potent angiogenic genes, including *Mmp10*, *Vegfa*, *Angiogenin 2*, *Nkx2.5*, *Gata-6*, and *PECAM-1* (CD31), that were drastically up-regulated in *Hipk1* ^−*/*−^ *;Hipk2* ^−*/*−^ mutants (Figure 2A). Indeed, immunohistochemistry using antibodies specific for VEGF-A, MMP10, or PAI-1 confirmed that these proteins were up-regulated in the endothelial cells of E9.5 *Hipk1* ^−*/*−^ *;Hipk2* ^−*/*−^ embryos (Figure 2B). In support of these results, qRT-PCR on *Vegf*, *Pai-1*, and *Mmp10* showed that the up-regulation of these genes was much more drastic in *Hipk1* ^−*/*−^ *;Hipk2* ^−*/*−^ mutant, but modest in *Hipk1* ^−*/*−^ *;Hipk2^+/+^* or *Hipk1^+/^* ^−^ *;Hipk2* ^−*/*−^ single mutants (Figure S1D), further supporting the cooperative role of HIPK1 and HIPK2 in the transcription of these targets.

### HIPK1 and HIPK2 Suppress MEF2C-Mediated Transcriptional Control of *Mmp10* and *Vegf* Expression

To further investigate the mechanisms of HIPK1/2, we focused on the transcription of *Mmp10* and *Vegf* because of their well-established functions in angiogenesis [2],[25]. Previous studies indicate that MEF2C promotes the transcription of *Mmp10* by binding to the upstream promoter. Interestingly, transcriptional corepressor HDAC7 suppresses MEF2C-dependent activation of *Mmp10* and that loss of HDAC7 leads to severe vascular phenotype and embryonic lethality similar to those in *Hipk1* ^−*/*−^ *;Hipk2* ^−*/*−^ mutants [25]. Since HIPK proteins have been implicated as transcriptional corepressors, we reasoned that HIPK1 and HIPK2 might suppress the transcription of *Mmp10* through its participation in the transcriptional complex involving HDAC7-MEF2C. Due to the role of HIPK2 in the TGF-β signaling pathway [13],[15], it is possible that HIPK1/2 may regulate *Mmp10* gene expression through Smad-dependent mechanisms. Alternatively, HIPK1/2 may function downstream of TGF-β downstream kinase, TAK1, which regulates vascular development during early embryogenesis [26]. Within the 1 kb upstream regulatory sequences of the *Mmp10* gene, we identified one Smad-binding element (SBE) site in position −221 to −215, close to the previously reported MEF2 recognition motif (TAAAATA) (position −80 to −73) (Figure 2C). Interestingly, however, unlike MEF2C, Smad2/3/4 by itself did not activate the transcriptional activity of *Mmp10-Luc* reporter (Figure S2). Rather, Smad2/3/4 modestly suppressed both wild-type *Mmp10-Luc* reporter and *Mmp10-Luc* mutating the SBE site (*Mmp10-mSBE-Luc*) (Figure S2), suggesting that the inhibitory effects of Smad2/3/4 on *Mmp10-Luc* reporter were most likely nonspecific. Furthermore, the presence of TGF-β did not change these results (Figure S2). In contrast to Smad2/3/4, MEF2C showed similar effects in promoting the transcriptional activity of wild-type *Mmp10-Luc* and *Mmp10-mSBE-Luc*, whereas mutating the MEF2-binding elements in *Mmp10*-luciferase reporter completely abolished the effects of MEF2C on this reporter (Figure 2D) [25]. These results supported the idea that the SBE site in the promoter of *Mmp10* was dispensable for MEF2C-mediated regulation of *Mmp10* gene expression, and that HIPK2 may regulate *Mmp10* transcription via MEF2C-dependent mechanism.

Consistent with its role as a transcriptional corepressor, HIPK2 showed a dose-dependent suppression of MEF2C-mediated activation of the *Mmp10-Luc* reporter (Figure 2E). The corepressor effects of HIPK2 required its kinase activity since the kinase inactive mutant HIPK2-K221A failed to suppress MEF2C-dependent activation of *Mmp10-Luc* reporter. Furthermore, the corepressor activity of HIPK2 required the protein–protein interaction domain (amino acids 582–898) because HIPK2 mutant protein lacking the C-terminal sequence from amino acid 898 to 1189 (HIPK2-Δ898) could still suppress *Mmp10-Luc* reporter, whereas further deletion from amino acid 582 to 1189 (HIPK2-Δ582) completely abolished the corepressor effects of HIPK2 (Figure 2E). Similar to HIPK2, HIPK1 could also suppress the MEF2C-dependent activation of *Mmp10-Luc* reporter. Although HIPK1 by itself was less effective compared to HIPK2 (unpublished data), HIPK1 and HIPK2 showed additive effects in suppressing the *Mmp10-Luc* activity (Figure 2F). To further characterize the transcriptional corepressor effects of HIPK2, we used siRNA to knock down the endogenous *Hipk2* expression in HEK293T cells and showed that lowering HIPK2 levels resulted in further up-regulation of MEF2C-mediated activation of *Mmp10*-*Luc* activity without affecting the levels of MEF2C (Figure 2G and Figure S3). Together, these results supported the novel role of HIPK1 and HIPK2 as transcriptional corepressors in MEF2C-mediated activation of *Mmp10* expression.

To determine if MEF2C and HIPK2 can also regulate the transcription of *Vegf*, we identified a potential MEF2 binding site in the *Vegf* locus (position −2679 to −2672) and generated a luciferase reporter that contained 4.5 Kb promoter sequence of *Vegf* gene (*Vegf-Luc*) (Figure 2H and Figure S4). Using similar approaches, we showed that MEF2C could indeed activate *Vegf-Luc* activity. Interestingly, MEF2C-mediated activation of *Vegf-Luc* could be suppressed by HIPK2 in a dose-dependent manner. Similar to the results from *Mmp10-Luc*, mutating the MEF2 binding element in *Vegf-Luc* reporter almost completely abolished the effects of MEF2C and HIPK2 (*mVegf-Luc*, Figure 2H). Furthermore, HIPK1 and HIPK2 also showed additive effects in suppressing the *Vegf-Luc* activity (Figure 2I). Although the effect of HIPK2 on *Vegf-Luc* reporter was not as robust as in *Mmp10-Luc*, these results were consistent with the previous results that the transcriptional controls of *Vegf* expression are a tightly regulated process such that loss of one *Vegf* allele or a slight increase in *Vegf* expression could result in marked abnormalities in angiogenesis during early embryonic development [27],[28].

### TGF-β Promotes Corepressor Complex Formation Between MEF2C and HIPK2 in the Regulation of *Mmp10* Expression

**Figure 3:**
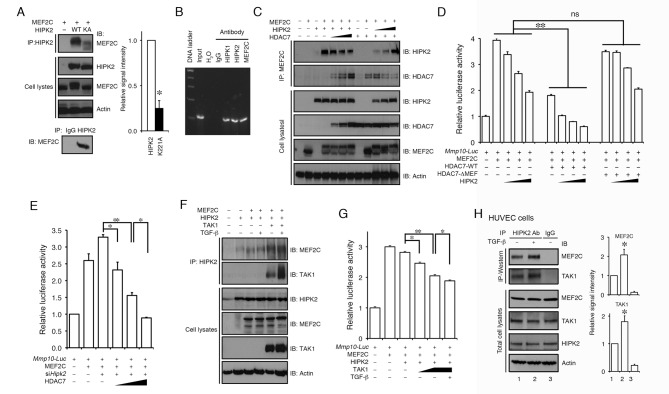
HIPK2 suppresses *Mmp10* expression through interaction with MEF2C and HDAC7. (A) Co-IP assays using protein lysates from HEK293T cells expressing HIPK2 and MEF2C show that HIPK2 can be detected in a protein complex with MEF2C (upper panels). Similar protein complex formation between endogenous HIPK2 and MEF2C can also be detected in *wild-type* MEF cells (lower panel). The interaction appears to depend on HIPK2 kinase activity as the kinase inactive HIPK2-K221A shows much reduced interaction with MEF2C. (B) ChIP assays using native chromatin from HUVECs show that HIPK1, HIPK2, and MEF2C can be detected in the promoter sequence of *Mmp10*. (C) Co-IP assays reveal that HIPK2 can be detected in a protein complex with HDAC7 and MEF2C. (D) HIPK2 and HDAC7 cooperatively suppress MEF2C-dependent activation of *Mmp10*-luciferase reporter activity. In the presence of the HDAC7 that lacks MEF2C interaction domain, HIPK2 can still suppress MEF2C in the activation of *Mmp10* reporter. (E) HDAC7 continues to suppress MEF2C-dependent activation of *Mmp10* in HEK293T cells where the endogenous *Hipk2* mRNA is reduced by siRNA. (F) Co-IP assays showing that TGF-β and TAK1 enhance the interaction between MEF2C and HIPK2. (G) TGF-β and TAK1 enhance the corepressor effects of HIPK2 on MEF2C-mediated *Mmp10* expression. (H) Co-IP assays using HUVEC cell lysates show that TGF-β promotes the interaction of endogenous HIPK2, TAK1, and MEF2C. Data are shown as mean ± s.e.m. Student's *t* test, *n* = 3 (*p<0.05, **p<0.01).

To further characterize the role of HIPK2 in the transcriptional control of *Mmp10* expression, we expressed MEF2C and HIPK2 in HEK293T cells and used co-immunoprecipitation (co-IP) to show that HIPK2 could indeed be detected in a complex with MEF2C (Figure 3A, upper panels). In addition, similar co-IP experiments using protein lysates from *wild-type* mouse embryonic fibroblasts (MEF) also showed that the endogenous HIPK2 proteins could be detected in a complex with MEF2C (Figure 3A, bottom panel). Consistent with the requirement of HIPK2 kinase activity in the transcriptional control of *Mmp10* (Figure 2E), the protein complex formation between kinase-inactive HIPK2-K221A and MEF2C was significantly reduced compared to *wild-type* HIPK2 (Figure 3A), whereas the MEF2C protein levels were comparable in cells expressing wild-type HIPK2 and kinase inactive HIPK2-K221A. The trace amount of MEF2C detected in the complex with HIPK2-K221A showed smaller molecular mass, suggesting that HIPK2 may affect the posttranslational modifications of MEF2C (Figure 3A). Indeed, treatment of alkaline phosphatase abolished the upward shift of MEF2C by HIPK2 (Figure S5), supporting the idea that the stable complex formation between HIPK2 and MEF2C required phosphorylation of MEF2C. To further characterize the involvement of HIPK2 and MEF2C in the regulation of *Mmp10* gene expression, we performed chromatin immunoprecipitation (ChIP) assays using native chromatin extracts from HUVEC and showed that endogenous MEF2C, HIPK1, and HIPK2 proteins were bound to the MEF2 site on the *Mmp10* promoter (Figure 3B). Similar results could also be detected in mouse brain microvascular endothelial (bEnd.3) cells (unpublished data).

Given that HDAC7 suppresses MEF2-mediated expression of *Mmp10* [25], we reasoned that HIPK2 might interact with the HDAC7-MEF2 transcriptional corepressor complex. Indeed, co-IP results using protein lysates from HEK293T cells overexpressing HIPK2, HDAC7, and MEF2C showed that HIPK2 and HDAC7 could each be detected in protein complexes with MEF2C (Figure 3C). Interestingly, however, the interaction between HIPK2 and MEF2C appeared to be reduced, but not completely eliminated, by the increasing amount of HDAC7. Conversely, the interaction between HDAC7 and MEF2 could also be reduced by the progressive increase in HIPK2 (Figure 3C). These results suggested that the recruitment of transcriptional corepressor complex to MEF2C might depend on the equilibrium between HIPK2 and HDAC7 [29]. Indeed, increasing the level of HIPK2 led to a progressive suppression of MEF2C-mediated activation of *Mmp10-Luc* reporter activity in the presence of HDAC7 (Figure 3D). To further determine if the corepressor activity of HIPK2 was dependent on HDAC7, we used a HDAC7 mutant that lacked the MEF2 interacting domain (HDAC7-ΔMEF) and therefore could not suppress MEF2-mediated transcription [25]. Interestingly, HIPK2 could suppress *Mmp10-Luc* activity in the presence of HDAC7-ΔMEF, suggesting that the transcriptional corepressor activity of HIPK2 could be independent of HDAC7 (Figure 3D). Consistent with these results, HIPK2 continued to suppress MEF2-mediated *Mmp10-Luc* activity in HEK293T cells in which the endogenous HDAC7 expression was reduced by siRNA (Figure S6). Similarly, HDAC7 could still suppress the *Mmp10-Luc* reporter activity in HEK293T cells treated with *Hipk2* siRNA (Figure 3E).

Several previous studies have indicated that HIPK2 and TAK1 cooperatively regulate the transcriptional activity of c-Myb through phosphorylation and proteasome-dependent degradation in the Wnt-1 signaling pathway [30],[31]. Since both TAK1 and HIPK2 have been implicated in the downstream of TGF-β [13],[32]–[34], we postulated that the transcriptional corepressor activity of HIPK2 might be further regulated by TAK1 in response to TGF-β. Consistent with this idea, co-IP assays showed that the presence of TAK1 and TGF-β enhanced the interaction between MEF2C and HIPK2 (Figure 3F). Moreover, the presence of TAK1 and TGF-β enhanced the corepressor effects of HIPK2 on MEF2C-mediated activation of the *Mmp10* luciferase reporter (Figure 3G). Consistent with these results, co-IP assays in HUVEC cells detected protein complex formation among endogenous HIPK2, TAK1, and MEF2C under normal growth conditions. Such interactions can be further promoted by treatment with TGF-β in HUVEC cells (Figure 3H).

### TGF-β and Its Downstream Kinase TAK1 Promote HIPK2 Phosphorylation

**Figure 4:**
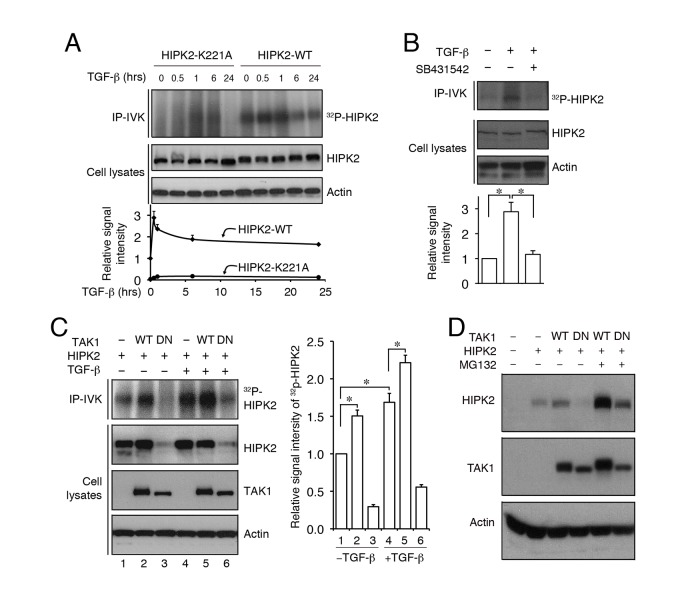
TGF-β–TAK1 promotes HIPK2 activity through protein–protein interaction and protects HIPK2 from proteasome-mediated degradation. (A) TGF-β promotes HIPK2 kinase activity in HEK293T cells, whereas kinase inactive HIPK2-K221A shows no incorporation of γ-^32^P-ATP upon TGF-β treatment. (B) The ability of TGF-β to activate HIPK2 kinase activity can be blocked by TGF-β type I receptor inhibitor SB431542. (C and D) TGF-β and *wild-type* TAK1 activate HIPK2 kinase and maintain the stability of HIPK2 protein. In contrast, dominant negative TAK1 (DN-TAK1) promotes HIPK2 degradation via the proteasome pathway.

The observation that mice lacking TAK1 exhibit severe vascular phenotype similar to *Hipk1* ^−*/*−^ *;Hipk2* ^−*/*−^ mutants [26] supports the idea that the protein complex involving HIPK2 and TAK1 may regulate TGF-β–dependent control of angiogenesis. To further characterize the role of HIPK2 in TGF-β signaling pathway, we performed immunoprecipitation–in vitro kinase (IP-IVK) assays and found that, under normal growth condition, HIPK2 showed a basal level of γ-^32^P-ATP incorporation. The addition of TGF-β further promoted the γ-^32^P-ATP incorporation in HIPK2 by 2- to 3-fold within 30′ to 1 h after treatment and remained higher than basal level for 24 h (Figure 4A). This effect was completely abolished in kinase-inactive HIPK2-K221A mutants or by TGF-β type I receptor ALK5 inhibitor SB431542 (Figure 4A,B). Since TAK1 has been shown to directly interact with TGF-β receptors [32],[34], we reasoned that the signal transduction from TGF-β to HIPK2 could induce a sequential activation of TAK1 and HIPK2 kinase activity through protein complex formation. Indeed, co-IP assays showed that TAK1 and HIPK2 formed a protein complex, and that the TAK1-HIPK2 complex formation could be further enhanced by TGF-β treatment (Figure 3F,H). These results were further supported by immunofluorescent confocal microscopy showing that TGF-β treatment promoted co-localization of HIPK2 and phospho-TAK1 in the nucleus of HUVEC cells (Figure S7). However, co-IP using TGF-β receptor antibodies showed protein complex formation between TGF-β receptors and TAK1, but not between TGF-β receptors and HIPK2 (unpublished data).

In addition to the interaction between TAK1 and HIPK2, our results showed that TAK1 could also activate the kinase activity of HIPK2. This effect was further enhanced by the treatment with TGF-β (Figure 4C). Surprisingly, expression of the dominant negative TAK1 (TAK1-DN), which carried a point mutation in the highly conserved lysine residue (K63W) in the kinase domain and therefore lacked kinase activity [35], led to a marked reduction in the HIPK2 protein level and HIPK2 kinase activity, even in the presence of TGF-β (Figure 4C). The effect of TAK1-DN on HIPK2 protein level appeared to be mediated by proteasome-dependent degradation since treatment with proteasome inhibitor MG-132 restored the level of HIPK2 protein in cells expressing TAK1-DN and further increased HIPK2 protein in cells expressing *wild-type* TAK1 (Figure 4D).

### TGF-β-TAK1 Signaling Phosphorylates HIPK2 on the Highly Conserved Tyrosine-361 Residue in the Kinase Domain

**Figure 5:**
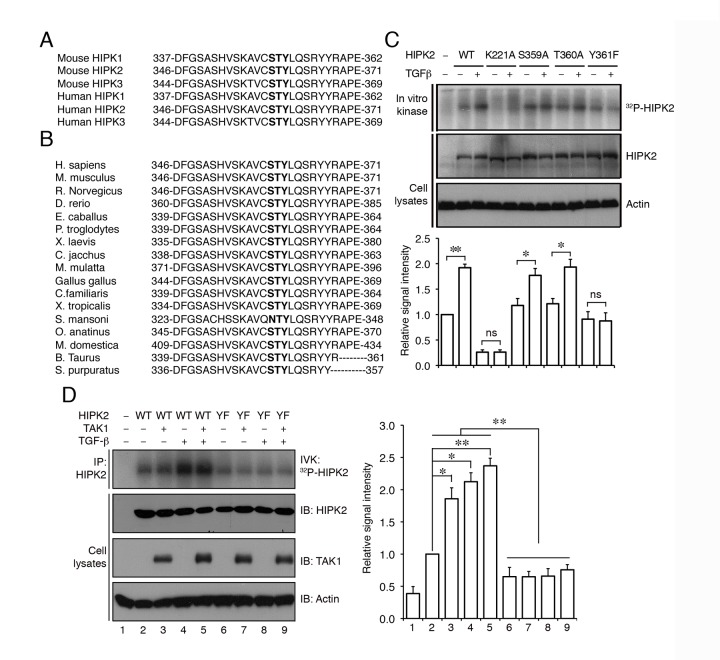
TGF-β activates HIPK2 by phosphorylating a highly conserved tyrosine residue on position 361. (A) Amino acid sequence alignment of the HIPK protein family from human and mouse reveals a stretch of highly conserved residues from position 346 to 371 in the activation segment of the subdomain VII in HIPK2. (B) Alignment of the similar regions of HIPK2 (346 to 371) from different species confirms that these amino acid residues are highly conserved from nematodes to the vertebrates. Conserved amino acids that can potentially be phosphorylated in MAPK signaling pathway are shown in bold. (C) The combined immunoprecipitation and in vitro kinase (IP-IVK) assays show that TGF-β treatment promotes the ability of *wild-type* HIPK2 to incorporate γ-^32^P-ATP. In contrast, kinase inactive HIPK2-K221A fails to incorporate γ-^32^P-ATP. While HIPK2-S359A and HIPK2-T360A mutant proteins can still incorporate γ-^32^P-ATP in response to TGF-β treatment, the Y361F mutation in HIPK2 completely eliminates its ability to incorporate γ-^32^P-ATP. (D) TGF-β and TAK1-induced phosphorylation of HIPK2 occurs primarily on Y361 residue in HIPK2. HIPK2-Y361F mutant completely loses its ability to incorporate γ-^32^P-ATP upon activation by TGF-β or TAK1. Data are shown as mean + s.e.m., *n* = 3. Statistics in (C) and (D) use Student's *t* test. *p<0.05, **p<0.01, ns = not significant.

The robust effects of TGF-β-TAK1 on HIPK2 phosphorylation raised the possibility that TGF-β could induce phosphorylation on specific amino acids in HIPK2 and thereby influence its transcriptional corepressor effects. Examinations of the amino acid sequence in the activation loop of the kinase domain of HIPK2 revealed a region from positions 346 to 371 that were highly conserved in HIPK1, HIPK2, and HIPK3 and among other species (Figure 5A,B). Since phosphorylation in the tripartite Ser-Thr-Tyr residues in positions 359, 360, and 361 of HIPK2 are similar to those identified in the activation loop of other MAP kinases [36],[37], we reasoned that TGF-β or TAK1 might promote phosphorylation on these amino acids in HIPK2. To address this, we mutagenized each of these amino acids and found that replacing S359 or T360 with a neutral amino acid did not affect the ability of HIPK2 to incorporate γ-^32^P-ATP (Figure 5C). In contrast, replacing Y361 with phenylalanine drastically reduced the ability of mutant HIPK2 (HIPK2-Y361F) to incorporate γ-^32^P-ATP upon activation by TGF-β or TAK1 (Figure 5C,D).

**Figure 6:**
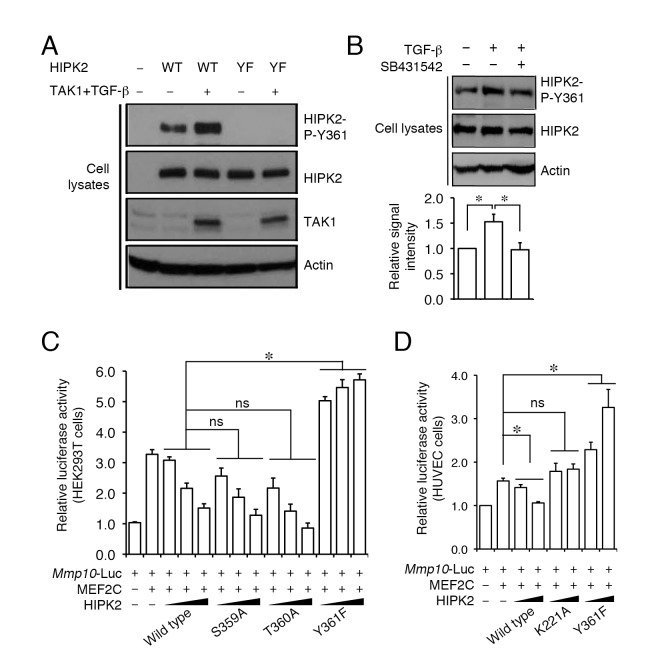
Mutation in Y361 of HIPK2 abolishes its ability to suppress *Mmp10-Luc* activity. (A) Phospho-specific antibody for HIPK2-P-Y361 confirms that TGF-β–TAK1 can indeed promote the phosphorylation of wild-type HIPK2 protein, but not HIPK2-Y361F mutant, in HEK293T cells. (B) TGF-β type I receptor (TβRI) inhibitor SB431542 blocks TGF-β–induced phosphorylation, but not basal phosphorylation, on Y361 residue in HIPK2 in HUVEC cells. (C and D) Phosphorylation on the Y361 residue of HIPK2 is required for the transcriptional suppressor effect of HIPK2 on *Mmp10* in HEK293T cells (C) and HUVEC cells (D). Data in (C) and (D) are shown as mean ± s.e.m., *n* = 3. Statistics in (C) and (D) use two-way ANOVA. *p<0.05, ns = not significant.

To further confirm that TGF-β-TAK1 promotes the phosphorylation of HIPK2 on Y361, we used a phospho-Y361–specific antibody (HIPK2-P-Y361) in Western blot analyses with cell lysates from HIPK2-TAK1–expressing HEK293T cells treated with or without TGF-β (Figure 6A). Similar to the results in Figure 5, we showed that, under normal growth conditions, HEK293T cells exhibited a steady-state level of HIPK2 phosphorylation on Y361, which could be further promoted by TGF-β (Figure 6A). In contrast, cells expressing HIPK2-Y361F mutant proteins showed no evidence of phosphorylated proteins that could be recognized by this antibody (Figure 6A). Interestingly, treatment with TGF-β inhibitor SB431542 completely abolished the effects of TGF-β, but did not affect the basal phosphorylation level of HIPK2-P-Y361 in HUVEC cells. These results suggested that additional TGF-β–independent mechanism(s) might regulate the basal phosphorylation of HIPK2-P-Y361 (Figure 6B).

To characterize the functional consequence of TGF-β–induced phosphorylation of HIPK2 on Y361, we performed *Mmp10-Luc* assays using wild-type HIPK2 and mutant HIPK2 with specific point mutation in the tripartite S359, T360, or Y361. Whereas HIPK2-S359A and HIPK2-T360A dose-dependently suppressed MEF2C-dependent activation of *Mmp10-Luc* just like wild-type HIPK2, this suppressor effect was completely abolished in HIPK2-Y361F (Figure 6C). These results were also confirmed in the HUVEC cells (Figure 6D). Together, these results indicated that TGF-β and TAK1 control the expression of angiogenic genes (e.g., *Mmp10*) by activating transcriptional corepressor HIPK2 via phosphorylation on a highly conserved tyrosine residue in the kinase domain.

### Perturbations of TGF-β Signaling in Endothelial Cells Recapitulates Angiogenesis Defects in *Hipk1* ^−*/*−^ *;Hipk2* ^−*/*−^ Mutants

**Figure 7:**
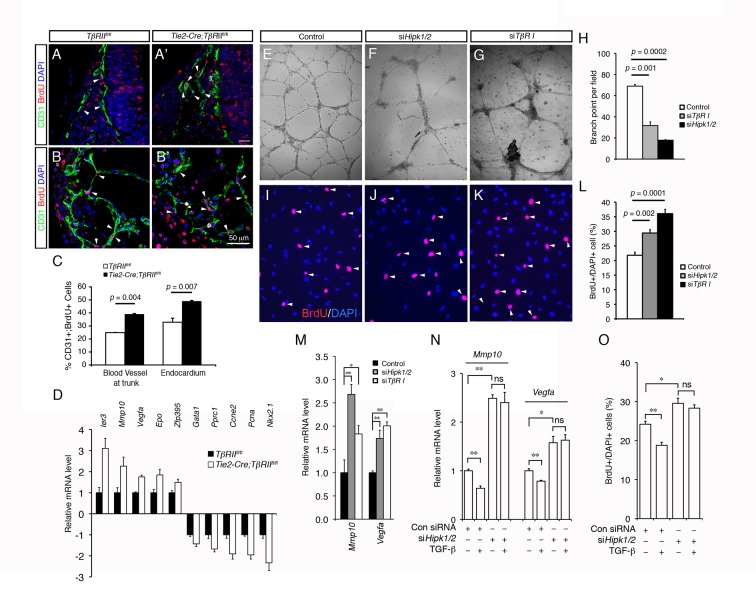
Perturbations of TGF-β signaling in endothelial cells recapitulate angiogenesis defects in *Hipk1* ^−^ ^***/*−**^ **;** ***Hipk2*** **^−^** ^***/*−**^ **mutants.** (A and B) Confocal images for the analyses of BrdU-incorporation in endothelial cells using anti-CD31 (green) and anti-BrdU (red) antibodies. Images are obtained from the blood vessels at the trunk region (panels A, A') and heart (panels B, B') of E9.5 control (*TβRII^fl/fl^*) and *Tie2-Cre;TβRII^fl/fl^* embryos. Arrows indicate CD31 and BrdU double positive cells. (C) Quantification of CD31 and BrdU double positive cells from blood vessels at the trunk level and the endocardium. Data are shown as mean ± s.e.m. Student's *t* test, *n* = 3. (D) qRT-PCR analyses show similar abnormalities in TGF-β target genes and angiogenic genes in *Tie2-Cre;TβRII^fl/fl^* embryos at E9.5. Data are shown as mean ± s.e.m, *n* = 3. (E–G) Matrigel assays show capillary-like structure formation in HUVECs treated with control siRNA, *Hipk1/2* siRNA, or *TβRI* siRNA. (H) Quantification of the number of branch points in Matrigel assays. (I–M) siRNA knockdown of *Hipk1/2* and *TβRI* promotes BrdU incorporation (I–L) and up-regulation of *Mmp10*, *Vegf*, and *Pai1* mRNA levels (M) in HUVEC cells. Arrowheads in panels (I–K) indicate BrdU+ cells. (N and O) The ability of TGF-β to suppress the expression of *Mmp10* and *Vegf* mRNA (panel N) and BrdU incorporation in HUVEC cells can be blocked by siRNA knockdown of *Hipk1/2*. Numbers in panels (H), (L), (M), and (N) are represented as means ± s.e.m. Student's *t* test, *n* = 3. *p<0.05, **p<0.01.

The results that HIPK2 can be activated by TAK1 in the TGF-β signaling pathway raised the possibility that endothelial cell-specific deletion of TGF-β signaling may result in phenotypes and perturbations in gene expression similar to those in *Hipk1* ^−*/*−^ *;Hipk2* ^−*/*−^ mutants. To test this, we generated conditional mutants that lacked TβRII in the endothelial cells by crossing the *TβRII^fl^* allele with the *Tie2-Cre*, which targets recombination in the endothelial cells as early as E7.5–8.5 in the developing embryos and yolk sacs [38]. Similar to *Hipk1* ^−*/*−^ *;Hipk2* ^−*/*−^ mutants, the *Tie2-Cre;TβRII^fl/fl^* mutants showed severe vascular defects and were lethal by E11.5–12.5. Analyses of the E9.5 *Tie2-Cre;TβRII^fl/fl^* mutant embryos showed a significant increase in the number of CD31+ endothelial cells in the trunk vasculature and in the developing endocardium (Figure 7A,B). The endothelial cells in *Tie2-Cre;TβRII^fl/fl^* mutants exhibited increases in BrdU incorporation (Figure 7C). Remarkably, qRT-PCR analyses of the mRNA from the E9.5 *Tie2-Cre;TβRII^fl/fl^* mutant embryos showed misregulations of TGF-β targets and angiogenesis genes similar to those seen in the *Hipk1* ^−*/*−^ *;Hipk2* ^−*/*−^ mutants (Figure 7D).

To further determine if loss of HIPK1 and HIPK2 or perturbations in TGF-β signaling recapitulates the vascular phenotype in *Hipk1* ^−*/*−^ *;Hipk2* ^−*/*−^ and *Tie2-Cre;TβRII^fl/fl^* mutants, we established in vitro angiogenesis assays using HUVEC cells cultured in growth-factor–reduced Matrigel to determine if siRNA knockdown of HIPK1 and HIPK2 (si*Hipk1/2*) or TGF-β type I receptor ALK5 (si*TβRI*) could affect vascular development in vitro. Our results indicated that HUVEC cells treated with control siRNA formed an intricate network of capillary-like structures (Figure 7E). In contrast, those treated with siRNA for *Hipk1*/*2* or *TβRI* showed poorly developed capillary-like structures and an increased propensity to form clusters of cells (Figure 7F–H), with a significant increase in BrdU incorporation (Figure 7I–L).

**Figure 8:**
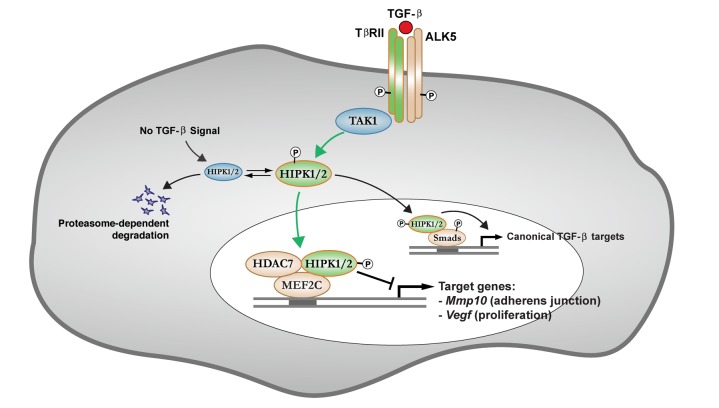
A working model for HIPK proteins in the transcriptional control of angiogenic gene expression in the downstream of TGF-β-TAK1 signaling pathway.

In addition to the Matrigel in vitro angiogenesis assays, we also examined the effects of TGF-β and HIPK1/2 in regulating the expression of *Mmp10* and *Vegf* genes and cellular proliferation in HUVEC cells. Using qRT-PCR, we showed that siRNA knockdown of *Hipk1/2* or *TβRI* led to up-regulations of *Mmp10* and *Vegf* mRNA levels in HUVEC cells (Figure 7M). In contrast, treatment of TGF-β suppressed the *Mmp10* and *Vegf* mRNA levels in HUVEC cells (Figure 7N). Interestingly, reducing HIPK1 and HIPK2 using siRNA blocked the ability of TGF-β to suppress the expression of *Mmp10* and *Vegf* (Figure 7N). Similar to these results, TGF-β–induced suppression of cellular proliferation in HUVEC cells, measured by BrdU incorporation, could also be blocked by siRNA knockdown of *Hipk1/2* (Figure 7O). Thus, the results from *Hipk1* ^−*/*−^ *;Hipk2* ^−*/*−^ mutants, *Tie2-Cre;TβRII* conditional mutants, the in vitro angiogenesis, and qRT-PCR assays in HUVEC cells supported the idea that the TGF-β–HIPK1/2 signaling pathway regulates a common set of target genes that are critical for angiogenesis during early embryonic development (Figure 8).

## Discussion

Perturbations to the TGF-β signaling mechanisms are known to have serious impacts on cardiovascular development in mice and in human diseases [7],[9]. The manifestations of mouse mutants with targeted deletion in TGF-β signaling components, however, are quite complex and, in some instances, seemingly conflicting. One possible contributing factor to such complexity is that different TGF-β receptors can trigger multiple, divergent downstream signaling via Smad and non-Smad-dependent mechanisms [12],[39]. In addition, the temporal and context-dependent effects of TGF-β on different cell types in the vasculature can further contribute to the final phenotypic outcomes [7]. TGF-β is known to either promote or antagonize endothelial proliferation and migration during vasculogenesis. Although the disparate outcomes of TGF-β are likely due to the differences in how TGF-β type I receptors ALK1 and ALK5 transduce its downstream signals, the exact mechanisms downstream of these receptors are not entirely clear [10],[11].

Our results reveal a previously unrecognized mechanism involving the cooperative role of HIPK1 and HIPK2 in the downstream of TGF-β–TAK1 signaling pathway that regulates the expression of a number of potent angiogenic genes during early embryonic development (Figure 8). First, based on the morphological analyses and gene expression profiling in *Hipk1* ^−*/*−^ *;Hipk2* ^−*/*−^ mutants, and the results from siRNA knockdown of *Hipk1* and *Hipk2* in Matrigel angiogenesis assays using HUVEC cells (Figures 1, 2, and 7), our data indicate that HIPK1 and HIPK2 act cooperatively to regulate a set of angiogenic genes, including *Mmp10* and *Vegf*, that are critical for the early stage of vascular development. This is further supported by a series of in vitro biochemical assays that validate HIPK2 and HDAC7 as important transcriptional corepressors that regulate the expression of *Mmp10* and *Vegf* (Figures 2 and 3). Consistent with these results, EM analyses also show that the endothelial cells in *Hipk1* ^−*/*−^ *;Hipk2* ^−*/*−^ mutants exhibit defects in the adherens junction formation similar to those described in *Hdac7* ^−*/*−^ mutants (Figure 1I–K) [25]. While HIPK2 and HDAC7 have synergistic effects in suppressing the transcription of *Mmp10*, each can work independently to suppress MEF2C-mediated gene expression. Surprisingly, the effect of HIPK2 and HDAC7 in MEF2C-mediated transcriptional control of *Mmp10* expression seems to depend on a delicate balance of protein–protein interaction in the transcriptional complex because increasing abundance of HIPK2 can reduce the presence of HDAC7 in complex with MEF2C and vice versa. One possible explanation for the antagonistic effect of HIPK2 and HDAC7 is that both may compete for the same or similar binding site in MEF2C, which can reach equilibrium as more HIPK2 or HDAC7 are recruited to the complex. This is particularly appealing because the transcriptional machinery involves dynamic assembly of large protein complexes that include transcriptional corepressors, such as HIPK2 and HDAC7 [29]. Alternatively, and not mutually exclusive, it is possible that HIPK2 and HDAC7 may cross-regulate each other through posttranslational modifications, such as phosphorylation or acetylation, which are likely to change the equilibrium of transcriptional complex formation.

The role of HIPK2 as a transcriptional corepressor of MEF2C proteins is further supported by the protein complex formation between MEF2C and HIPK2 in HEK293T cells. Such protein complex formation between endogenous HIPK2 and MEF2C can also be detected in *wild-type* MEF and HUVEC cells (Figure 3). Interestingly, the interaction between HIPK2 and MEF2C seems to require the kinase activity of HIPK2 because significantly fewer MEF2C proteins are detected in a complex with kinase inactive HIPK2-K221A. Furthermore, the MEF2C proteins that do interact with HIPK2-K221A have lower molecular mass compared with those in complex with *wild-type* HIPK2, suggesting that HIPK2 may posttranslationally modify MEF2C and thereby inhibits the transcriptional activity of MEF2C. In support of this idea, alkaline phosphatase treatment reduces the HIPK2-induced high molecular mass migration of MEF2C in SDS-PAGE (Figure S5). Although there is no evidence that MEF2C is a direct phosphorylation substrate for HIPK2, it is possible that HIPK2 may activate other protein kinases, such as Cdk5 and GSK3β [40],[41], to phosphorylate MEF2 and thereby promote the pro-differentiation function of MEF2 in endothelial cells.

One remarkable finding from this study is the identification of TGF-β and TGF-β–activating kinase 1 (TAK1) as upstream mechanisms that regulate the interaction between HIPK2, HDAC7, and MEF2C (Figures 3 and 4). These results indicate that TAK1 have two distinct roles in regulating HIPK2 functions. First, using immunoprecipitation–in vitro kinase (IP-IVK) assays, we show that both TGF-β and TAK1 can activate HIPK2 by phosphorylating the tyrosine on position 361 (Y361), a highly conserved residue among all HIPK members in the activation loop of the kinase domain (Figure 5). These results are further verified using a phospho-HIPK2 specific antibody, HIPK2-P-Y361 (Figure 6). Strikingly, HIPK2 with a tyrosine-to-phenylalanine mutation (HIPK2-Y361F) on this amino acid completely loses its ability to suppress MEF2C-dependent transcriptional activity (Figure 6). Second, and quite unexpectedly, we discover that kinase inactive TAK1 blocks HIPK2 function by promoting the degradation of HIPK2 through proteasome-dependent mechanisms (Figure 4). Consistent with these results, treatment with TAK1 inhibitor 5Z-7-Oxozeaenol also promotes HIPK2 degradation in HEK293T cells (Y.S., unpublished observations). These results suggest that, in the absence of signal from TGF-β–TAK1, dephosphorylated HIPK2 proteins may undergo rapid turnover via proteasome pathway (Figure 8). Alternatively, kinase inactive TAK1 may alter intracellular transport of HIPK2 and promote proteasome-mediated degradation of HIPK2. Given the closely interconnected functions between TAK1 and HIPK2, it is perhaps not surprising that loss of TAK1 results in early embryonic lethality due to defects in vascular morphogenesis similar to those in *Hipk1* ^−*/*−^ *;Hipk2* ^−*/*−^ mutants [26].

While our results highlight the robust effects of HIPK1 and HIPK2 as corepressors in the MEF2C-dependent transcriptional activation of angiogenic genes, there are several indications that HIPK proteins may have broader functions in regulating the outcome of TGF-β signaling. For instance, HIPK2 has been shown to serve as a transcriptional coactivator in the Smad2/3/4-SBE reporter assays and in JNK-mediated functions, which critically regulate the decision of survival and apoptosis in dopaminergic neurons and in tumor cells, respectively [13],[16]. In addition, HIPK2 can also function as a corepressor in Ski-dependent suppression of BMP-Smad1/4-induced transcriptional activation [15]. Given the complexity of TGF-β signaling mechanisms, it is possible that the final outcomes of HIPK2 functions will likely be context-dependent. In support of this view, loss of HIPK1 and HIPK2 leads to down-regulation of several genes critical for the control of cell cycle progression (Figures 2 and 8). Although the magnitudes of reduction in these genes are not as drastic as the up-regulation of angiogenic genes, many of these genes have been well-documented to be the transcriptional targets in the canonical TGF-β–Smad pathway (Figure 8 and Table S1) [42]. It will be interesting to determine if HIPK1 and HIPK2 may regulate the transcriptional control of these target genes, thus establishing these kinases as novel mediators connecting the Smad and non-Smad signaling pathways downstream of TGF-β. Finally, the gene expression data in *Hipk1* ^−*/*−^ *;Hipk2* ^−*/*−^ mutants also reveal a significant, albeit modest, down-regulation of *Alk1*, *Alk5*, and *Hdac7* transcripts. While it is unclear if HIPK1 and HIPK2 can also directly regulate the transcription of these genes, based on the well-characterized functions of these genes, their down-regulation could certainly amplify the vascular defects in *Hipk1* ^−*/*−^ *;Hipk2* ^−*/*−^ mutants.

## Materials and Methods

### Mice

The *Hipk1* ^−*/*−^ and *Hipk2* ^−*/*−^ mutant mice have been described previously [18],[43]. The *Tie2-Cre* and the floxed TGF-β type II receptor (*TβRII^fl^*) mice were generously provided by Dr. Rong Wang and Dr. Harold Moses, respectively [38],[44],[45]. Animal care was approved by the Institutional of Animal Care and Use Committee and followed the NIH guidelines.

### Immunohistochemistry, Fluorescence Microscopy, and Electron Microscopy

Embryonic day (E) 9.5 and E10.5 embryos and yolk sacs were fixed at 1% PFA in PBS for 2 h, cryoprotected in 15% sucrose for 30 min, and then in 30% sucrose for 30 min. Tissue sections were incubated with primary antibodies overnight and with secondary antibodies for 1 h. To label the cells in S-phase of cell cycle, pregnant mice were injected intraperitoneally with BrdU (50 mg/kg body weight, BD Bioscience) and sacrificed 2 h later. To detect BrdU+ endothelial cells, tissue sections were incubated with the CD31 antibody. Afterward, the tissue sections were fixed in 4% PFA for 30 min and then treated with 2N HCl at 37°C for 30 min. After three washes with Borax solution, the tissue sections were incubated with primary antibody against BrdU overnight, and then incubated with Alexa Fluor-conjugated secondary antibody for 1 h. For whole-mount immunofluoescent staining, E9.5 embryos and yolk sacs were fixed in 4% PFA in PBS overnight at 4°C, washed four times in PBS at 4°C, and blocked overnight at 4°C in 5% goat serum, 0.1% Triton X-100 in PBS. They were then incubated in rat anti-CD31 antibody (1∶500; Mec13.3; BD Biosciences) overnight at 4°C, washed in PBT overnight at 4°C, and incubated in Alexa Fluor 488 Goat Anti-Rat IgG (1∶1,000) for embryos and Alexa Fluor 555 Goat Anti-Rat IgG (1∶2,000) for yolk sacs. To determine the number of endothelial cells in S-phase of the cell cycle, tissue sections were double labeled with anti-CD31 (1∶20; Cat No. 550274; BD Biosciences) and anti-BrdU (1∶500; MAB3222; Millipore). Immunohistochemistry using PAI1 antibodies required antigen retrieval, in which the tissue sections were incubated in 10 mM sodium citrate buffer at 100°C for 30 min. Sample preparations and image capture for electron microscopy were described previously [14].

### Quantification of Vascular Development in Yolk Sacs

Neurolucida was used to determine the avascular area, fragment length (length of a vessel before it branches), and branch points in the yolk sacs. Individual avascular areas were manually traced and then added up to get the total avascular area per frame using “contour mapping” option in Neurolucida (MicroBrightField). Individual fragment lengths were measured with each fragment length separated by a different colored line. Fragment lengths were then averaged to get the average fragment length per frame [46].

### RT-PCR and Microarray Analyses

Total RNA was extracted from embryos using PicoPure RNA Isolation Kit (Arcturus) and used as a template for reverse transcriptase with MessageAmp II-Biotin enhanced Kit (Ambion). Microarray analysis was performed using CodeLink Mouse Whole Genome Bioarray (Applied Microarrays). The microarray data have been deposited in Gene Expression Omnibus (http://www.ncbi.nlm.nih.gov/geo/), accession number GSE39253. The RNA from HEK293T cells, HUVEC cells, or MEF was isolated by Trizol reagent (Invitrogen) and used as a template for reverse transcriptase with random hexamer primers (Invitrogen). Primer sequences for specific genes are available in Table S2.

### Cell Cultures

HEK293T cells were purchased from ATCC and MEFs was reported previously [47]. Both cell lines were cultured in DMEM growth medium with 10% fetal bovine serum (Hyclone). HUVEC cells were maintained in EGM-2 medium (Lonza Walkerville Inc.). For immunostaining, cells were plated on gelatin-coated glass coverslips, fixed in 4% PFA, and stained with appropriate primary antibodies as described previously [43],[47].

### siRNA and Luciferase Reporter Assays

siRNA oligonucleotides for *Hipk1* (Cat No. sc39048), *Hipk2* (Cat No. sc39050), *Hdac7* (Cat No. sc35546), or TGF-β type I receptor (*TβRI*) (Cat No. sc40222, specific for *ALK5*) were purchased from Santa Cruz Biotechnology, Inc. and used at a concentration of 30 pM to transfect HEK293T or HUVEC cells using Lipofectamine 2000 (Invitrogen). Two days after transfection, cells were harvested either for RNA isolation or for luciferase activity measurement. RT-PCR and Western blots were performed multiple times with comparable results. Primer sequences for PCR were provided in Table S2. Luciferase assays were performed using the dual-luciferase assay system (Promega) [13],[43],[47]. The luciferase reporter activity was measured using the dual-luciferase system on a luminometer (Turner Designs). Relative luciferase activity was reported as a ratio of firefly over Renilla luciferase readouts. The *Mmp10*-luciferase reporters, HDAC7 constructs, and myc-tagged MEF2C construct were gifts from Dr. E. Olson [25]. The *Vegfa*-luciferase reporter contained 4,512 bp to 1 bp of the mouse *Vegfa* gene, subcloned into pGL4.10[*Luc2*] vector (Promega). The *Vegfa*-luciferase construct that contained mutations in the MEF2 binding site (*mVegfa-luc*) was generated using the QuikChange II Site-Directed Mutagenesis kit (Stratagene).

### Co-IP and In Vitro Kinase Assay

Whole-cell lysates were collected from HEK293T cells 24 h after transfection in lysis buffer containing 50 mM HEPES (pH 7.4), 50 mM NaCl, 0.1% Tween 20, 20% glycerol, and 1× protease inhibitor cocktail (Roche Molecular Systems) with brief sonication. The same amount of supernatants was incubated overnight at 4°C with different primary antibody and then incubated with Protein A/G Plus Agarose beads for 3 h at 4°C. Immune complexes were washed in buffers containing 50 mM HEPES (pH 7.4), 300 mM NaCl, 0.2 mM EDTA, and 1% NP-40 and analyzed on SDS/PAGE. For in vitro kinase assays, cells were treated with DMSO or 10 ng/ml TGF-β 24 h after transfection, and then whole-cell lysates were collected in lysis buffer. Immune complexes were washed with kinase buffer (25 mM Tris-HCl, pH 8.0, 10 mM MgCl_2_), and then incubated with 1 mM ATP and 5 µCi of γ-^32^P-ATP (Perkin Elmer) for 3 h at room temperature. The resin beads were then washed with 10 nM Tris-HCl (pH 7.5) and the proteins eluted with 25 µl SDS loading buffer. Phosphorylation of HIPK2 on Y361 was confirmed by HIPK2-P-Y361 specific antibody (Thermo Scientific, Cat No. PA5-13045, 1∶500 dilution) in Western blots using HEK293T cell lysates.

### ChIP Assay

ChIP assays were performed as described [47]. Briefly, HUVEC or bEnd.3 cells were fixed with 4% PFA and treated with SDS lysis buffer. After shearing with a sonicator and contrifugation, the supernatant of cell lysates were used for immunoprecipitation with different antibodies. The DNA–protein–antibody complexes were isolated using antibodies for HIPK1 (p-16, sc-10289), MEF2C (e-17, sc-13266) (Santa Cruz Biotechnology), or HIPK2 (ab28507, Abcam). The complexes were washed with buffers, and the DNA were eluted and purified. Primer sequences were available in Table S2.

### In Vitro Angiogenesis and BrdU Incorporation Assays

HUVEC cells were cultured in EBM-2 medium containing serum and endothelial cell supplements (EGM2) according to the manufacturer's instructions (BD Biosciences). The siRNA-mediated knockdown was performed when the cells reached 80% confluence. For in vitro angiogenesis assays, HUVEC cells were trypsinized 48 h after transfection, and reseeded onto Matrigel-coated plate in the presence of EGM2 medium. After 18 h, vascular formation was assessed and photographed under a Nikon TE2000-U microscope with 4× objective. For BrdU incorporation assays, HUVECs were seeded onto gelatin-coated coverslips in 24-well plates, and incubated with BrdU (10 µM) for 2.5 h.

### Statistical Analyses

Data were analyzed by two-tailed Student's *t* test. Values were expressed as mean ± S.E.M. Changes were identified as significant if the *p* value was less than 0.05.

## Supporting Information

Figure S1 (A) Unsupervised hierarchical clustering analyses of all clones with a standard deviation >0.01 in expression levels. The gene expression profiles in *Hipk1* ^−*/*−^ embryos are more related to those in *wild-type*, whereas those in *Hipk2* ^−*/*−^ embryos are more related to *Hipk1* ^−*/*−^ *;Hipk2* ^−*/*−^ embryos. (B) Gene Ontogeny and KEGG pathway analyses indicated that only a very small number of genes in *Hipk1* ^−*/*−^ embryos showed altered expression patterns. In contrast, the number of affected genes showed a progressive increase in *Hipk2* ^−*/*−^ and *Hipk1* ^−*/*−^ *;Hipk2* ^−*/*−^ mutants. (C) Quantitative analyses of the perturbations of TGF-β target genes using microarrays and qRT-PCR analyses. (D) Quantitative RT-PCR analyses of three representative angiogenic genes, *Vegf*, *Mmp10*, and *Pai1*, confirm the cooperative role of HIPK1 and HIPK2 in regulating the expression of these targets. Data are shown as mean ± s.e.m. Student's *t* test, *n* = 3.
(TIF)

Figure S2 Mutation of the Smad-binding element (SBE) in the promoter of *Mmp10* does not affect MEF2C-mediated regulation of *Mmp10-Luc* activity. MEF2C has similar effects in activating the luciferase activity of wild-type *Mmp10-Luc* or Mmp10-Luc mutating the SBE (*Mmp10-mSBE-Luc*). In contrast, Smad2/3/4 does not affect *Mmp10-Luc* or *Mmp10-mSBE-Luc* activity either with or without TGF-β. Although Smad2/3/4 appears to suppress *Mmp10-Luc* and *Mmp10-mSBE-Luc* activity, this is likely to be a nonspecific or indirect effect.
(TIF)

Figure S3 Knockdown of *Hipk2* mRNA level using siRNA does not affect MEF2C protein level.
(TIF)

Figure S4 A schematic diagram indicating that the mouse *Vegf* locus contains seven coding exons. Sequence analyses of the 4,512-bp regulatory sequence upstream to the ATG of the first coding exon reveal a MEF2 binding element (TAAAAATA) from position −2,679 to −2,672. The 4,512-bp regulatory element is used to generate two *Vegf*-luciferase reporter constructs, one with the *wild-type* MEF2 binding site and the other with the MEF2 binding element mutated to TAGGGGTA.
(TIF)

Figure S5 Alkaline phosphatase treatment reduces the high molecular mass migration of MEF2C in SDS-PAGE, suggesting that HIPK2 may promote phosphorylation in MEF2C.
(TIF)

Figure S6 HIPK2 continues to suppress MEF2C-mediated activation of *Mmp10*-luciferase activity in HEK293T cells in which the endogenous HDAC7 is knocked down by siRNA.
(TIF)

Figure S7 TGF-β promotes co-localization of HIPK2 and activated TAK1 (pTAK1) in the nuclei of HUVEC cells.
(TIF)

Table S1 TGF-β target genes that are up- or down-regulated in *Hipk1* ^−*/*−^; *Hipk2* ^−*/*−^ mutants.
(DOC)

Table S2 List of primer sequences (5′ to 3′) used in this study.
(DOC)
