# Supplementary figures and images for: Transcriptional Corepressors HIPK1 and HIPK2 Control Angiogenesis Via TGF-β–TAK1–Dependent Mechanism
Source: PLoS Biol. 2013 Apr 2;11(4):e1001527. doi: 10.1371/journal.pbio.1001527 (PMC3614511; doi:10.1371/journal.pbio.1001527)

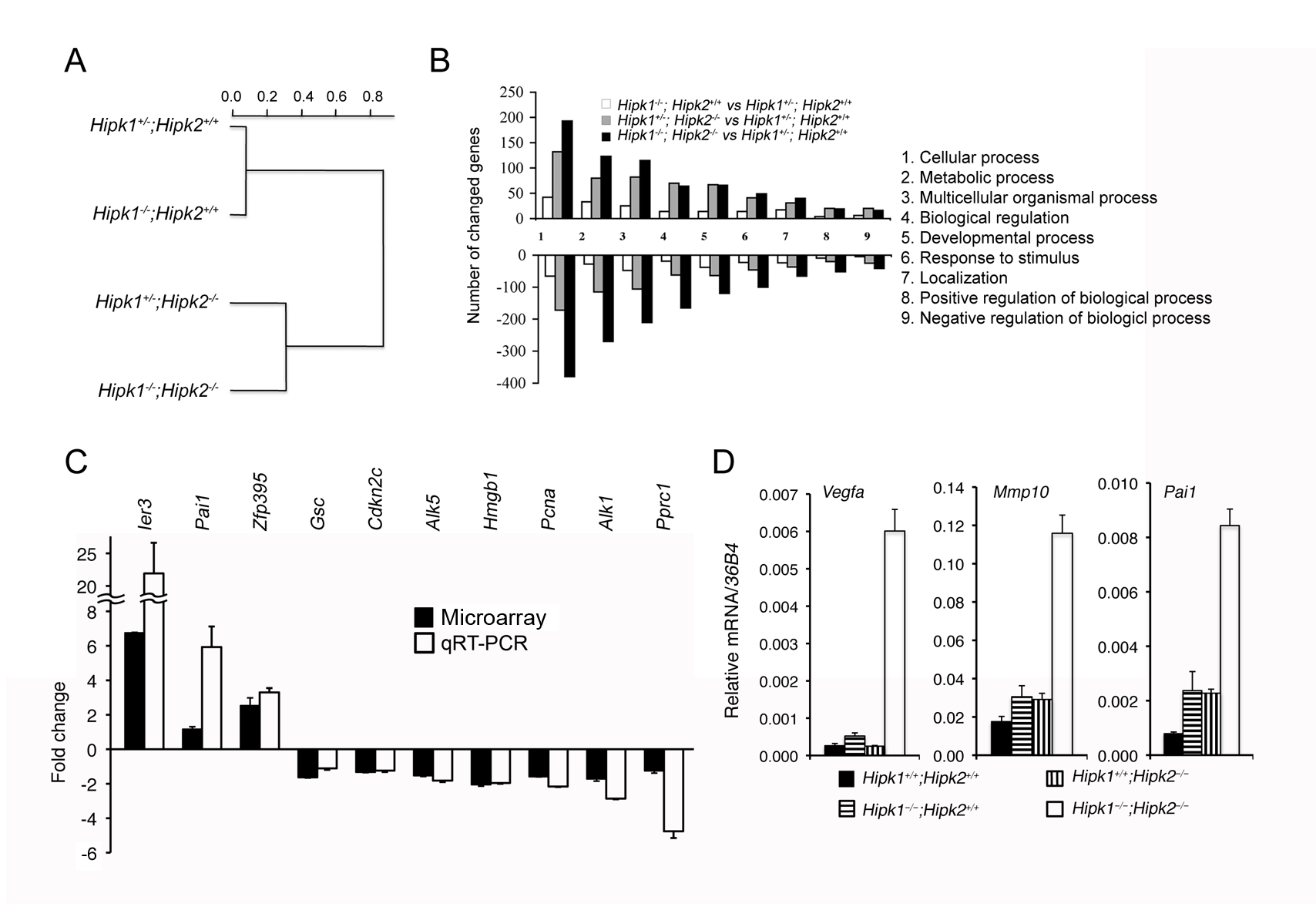

Supplement: Figure S1 — (A) Unsupervised hierarchical clustering analyses of all clones with a standard deviation >0.01 in expression levels. The gene expression profiles in Hipk1 −/− embryos are more related to those in wild-type, whereas those in Hipk2 −/− embryos are more related to Hipk1 −/− ;Hipk2 −/− embryos. (B) Gene Ontogeny and KEGG pathway analyses indicated that only a very small number of genes in Hipk1 −/− embryos showed altered expression patterns. In contrast, the number of affected genes showed a progressive increase in Hipk2 −/− and Hipk1 −/− ;Hipk2 −/− mutants. (C) Quantitative analyses of the perturbations of TGF-β target genes using microarrays and qRT-PCR analyses. (D) Quantitative RT-PCR analyses of three representative angiogenic genes, Vegf, Mmp10, and Pai1, confirm the cooperative role of HIPK1 and HIPK2 in regulating the expression of these targets. Data are shown as mean ± s.e.m. Student's t test, n = 3. (TIF) [file pbio.1001527.s001.tif]

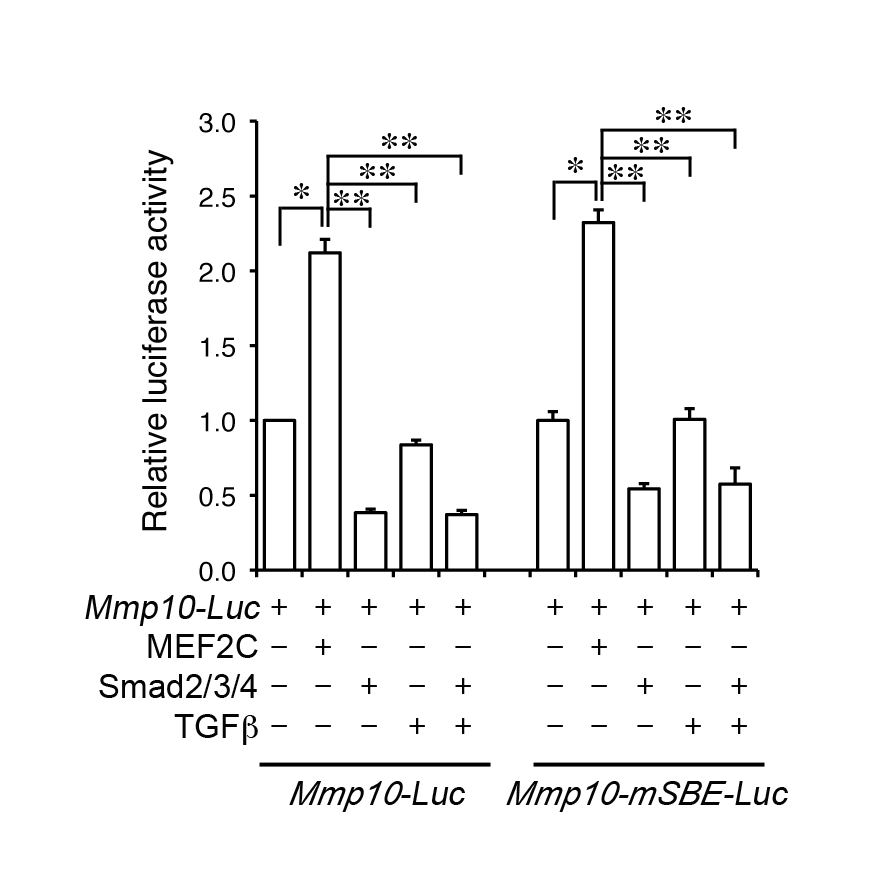

Supplement: Figure S2 — Mutation of the Smad-binding element (SBE) in the promoter of Mmp10 does not affect MEF2C-mediated regulation of Mmp10-Luc activity. MEF2C has similar effects in activating the luciferase activity of wild-type Mmp10-Luc or Mmp10-Luc mutating the SBE (Mmp10-mSBE-Luc). In contrast, Smad2/3/4 does not affect Mmp10-Luc or Mmp10-mSBE-Luc activity either with or without TGF-β. Although Smad2/3/4 appears to suppress Mmp10-Luc and Mmp10-mSBE-Luc activity, this is likely to be a nonspecific or indirect effect. (TIF) [file pbio.1001527.s002.tif]

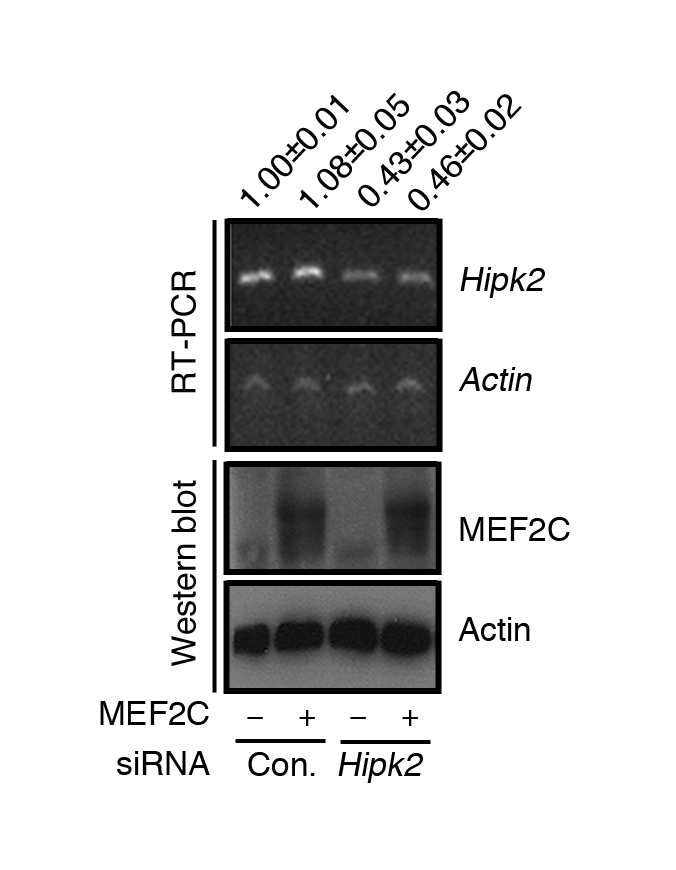

Supplement: Figure S3 — Knockdown of Hipk2 mRNA level using siRNA does not affect MEF2C protein level. (TIF) [file pbio.1001527.s003.tif]

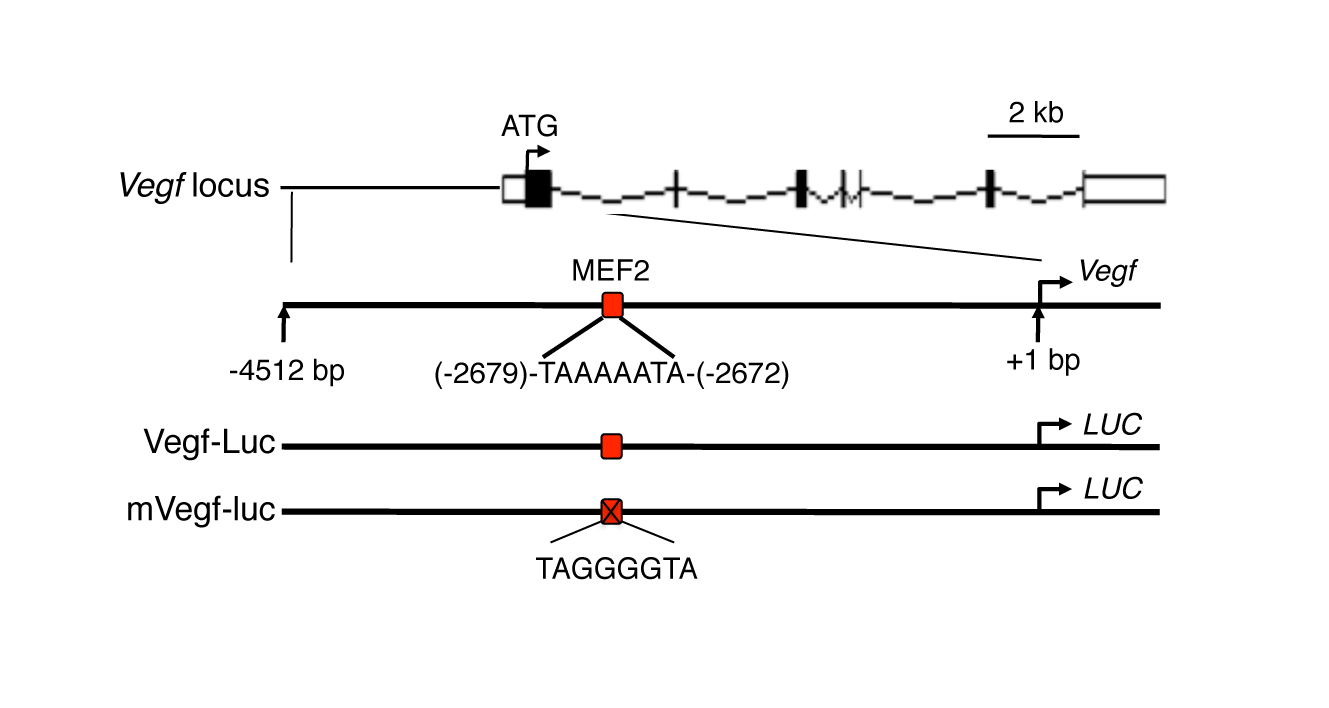

Supplement: Figure S4 — A schematic diagram indicating that the mouse Vegf locus contains seven coding exons. Sequence analyses of the 4,512-bp regulatory sequence upstream to the ATG of the first coding exon reveal a MEF2 binding element (TAAAAATA) from position −2,679 to −2,672. The 4,512-bp regulatory element is used to generate two Vegf-luciferase reporter constructs, one with the wild-type MEF2 binding site and the other with the MEF2 binding element mutated to TAGGGGTA. (TIF) [file pbio.1001527.s004.tif]

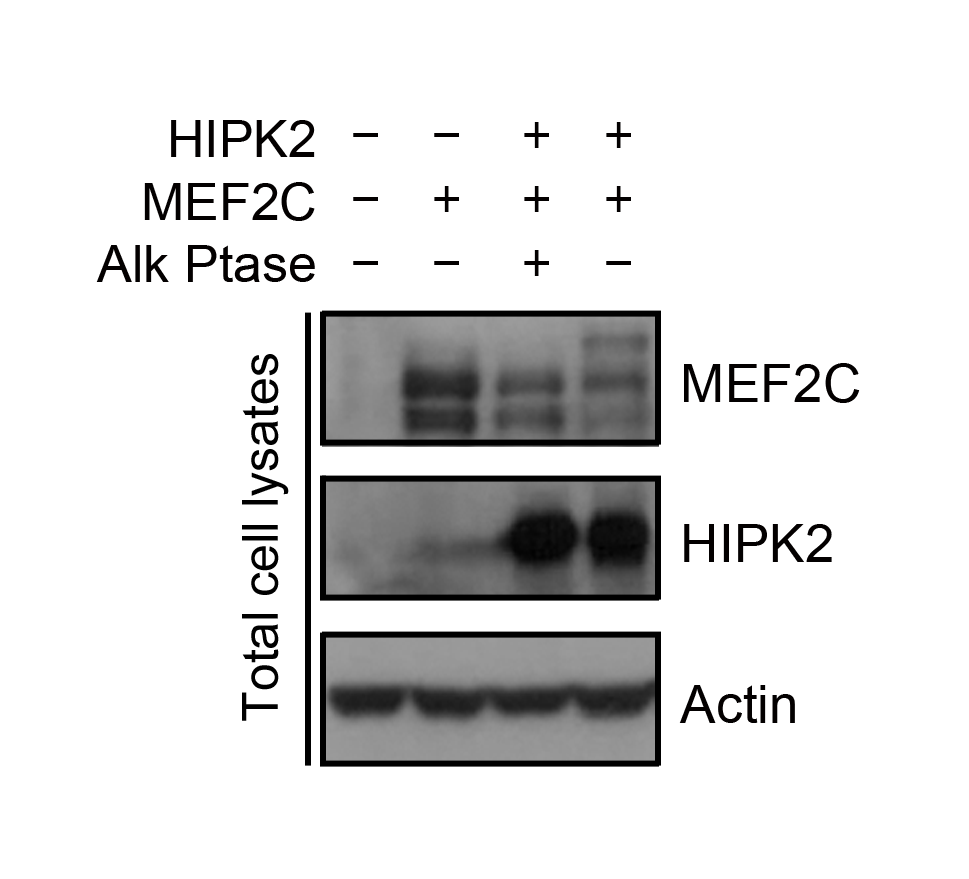

Supplement: Figure S5 — Alkaline phosphatase treatment reduces the high molecular mass migration of MEF2C in SDS-PAGE, suggesting that HIPK2 may promote phosphorylation in MEF2C. (TIF) [file pbio.1001527.s005.tif]

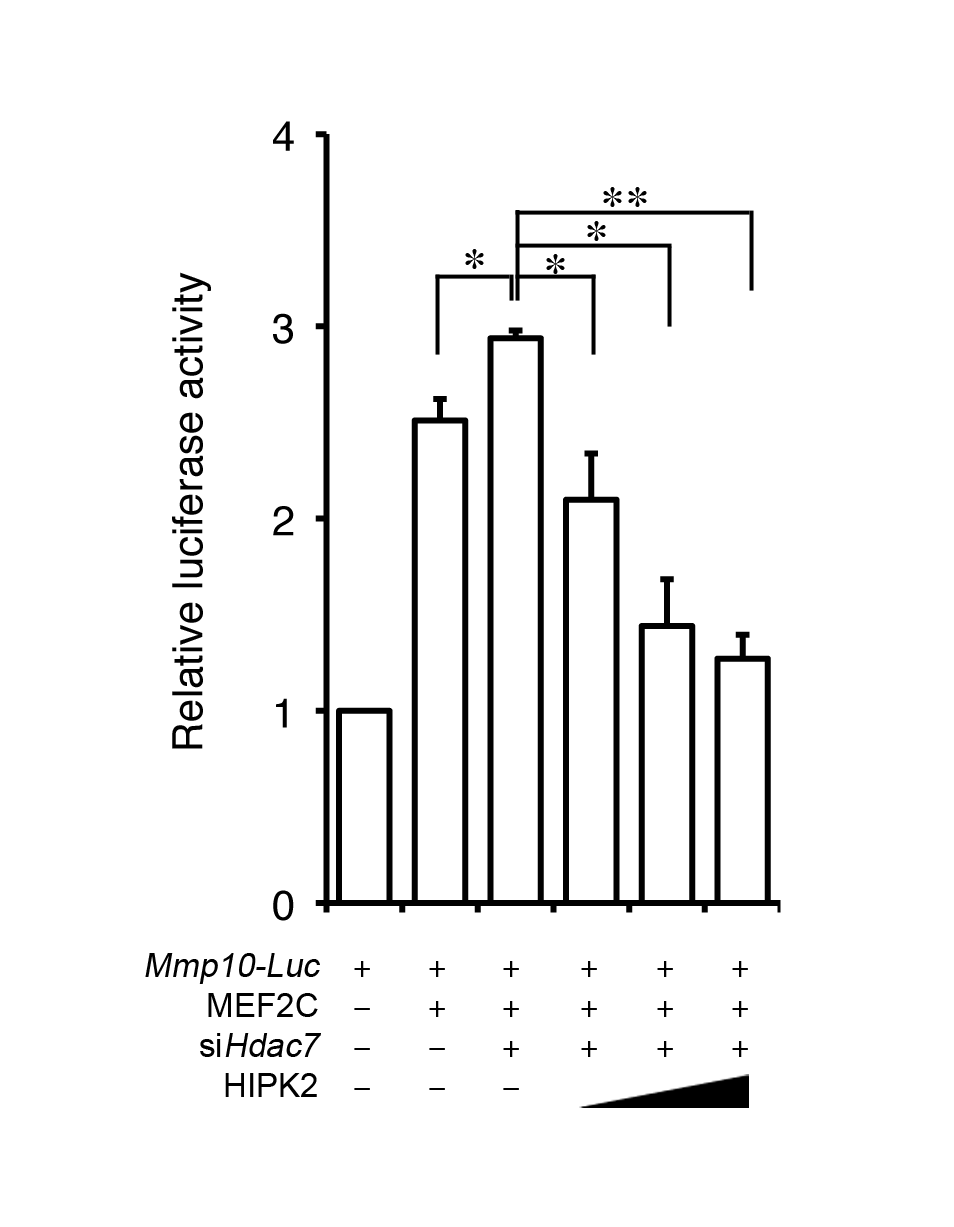

Supplement: Figure S6 — HIPK2 continues to suppress MEF2C-mediated activation of Mmp10-luciferase activity in HEK293T cells in which the endogenous HDAC7 is knocked down by siRNA. (TIF) [file pbio.1001527.s006.tif]

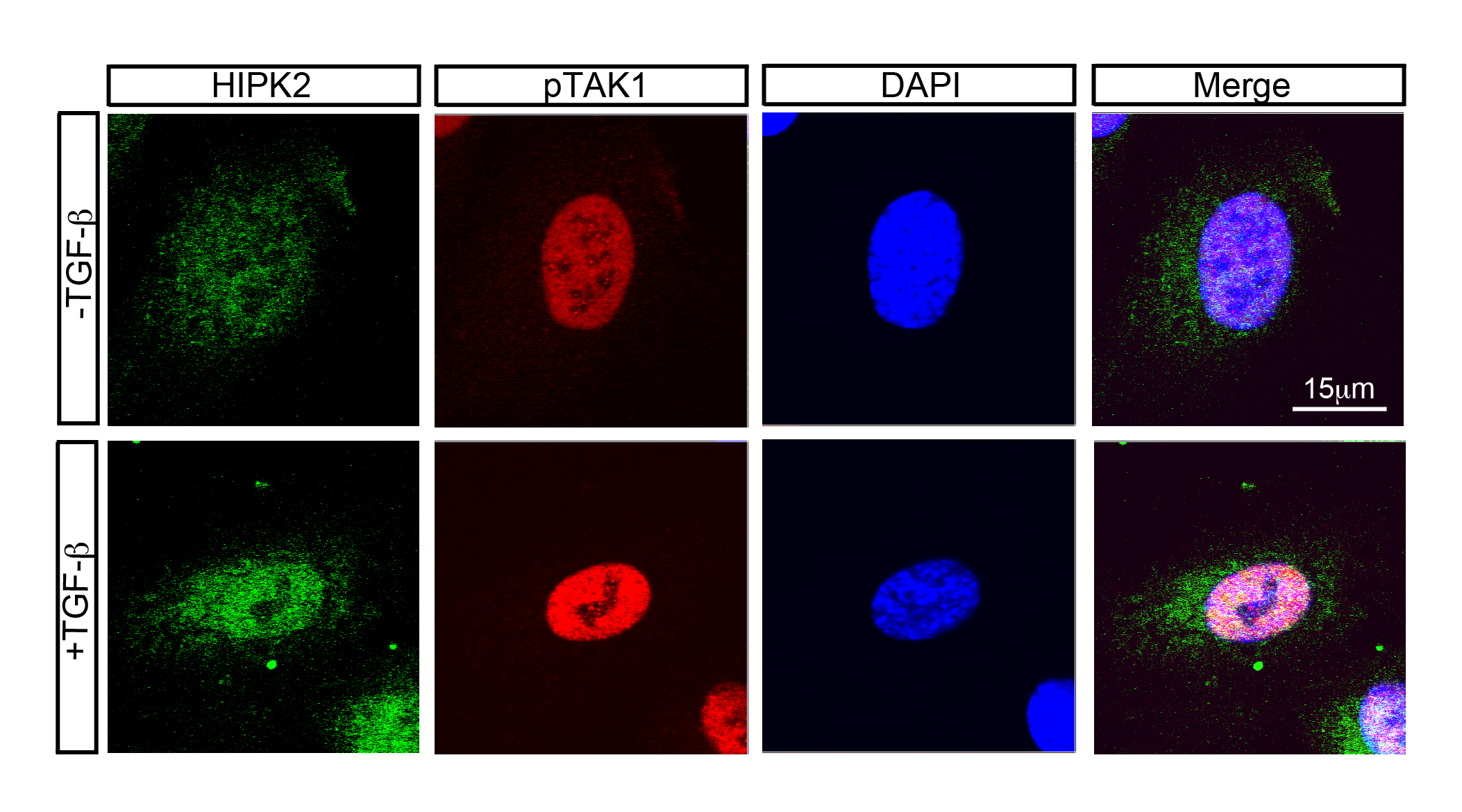

Supplement: Figure S7 — TGF-β promotes co-localization of HIPK2 and activated TAK1 (pTAK1) in the nuclei of HUVEC cells. (TIF) [file pbio.1001527.s007.tif]
