# Supplementary material for: Transcriptional Corepressors HIPK1 and HIPK2 Control Angiogenesis Via TGF-β–TAK1–Dependent Mechanism
Source: PLoS Biol. 2013 Apr 2;11(4):e1001527. doi: 10.1371/journal.pbio.1001527 (PMC3614511; doi:10.1371/journal.pbio.1001527)
Supplement: Table S1 — TGF-β target genes that are up- or down-regulated in Hipk1 −/−; Hipk2 −/− mutants. (DOC) [file pbio.1001527.s008.doc]

| **Gene Ontology (GO) sorting to TGF-β signaling** | | | | | |
| --- | --- | --- | --- | --- | --- |
| **WT** | **dKO** | **Ratio** | **p-value** | **Gene ID** | **Gene Name** |
| 0.157 | -0.644 | 1.74 | 0.0307 | NM_016769 d | MAD homolog 3 (Drosophila) |
| 3.351 | 2.589 | 1.70 | 0.0242 | NM_010849 d | Myelocytomatosis oncogene * |
| 0.484 | -0.248 | 1.66 | 0.0012 | NM_009758 d | Bone morphogenetic protein receptor, type 1A |
| -0.054 | -0.779 | 1.65 | 0.0232 | NM_009370 d | Transforming growth factor, beta receptor I |
| 2.280 | 1.651 | 1.55 | 0.0110 | NM_012042 d | Cullin 1 |
| 0.293 | 1.699 | 2.65 | 0.0483 | NM_199029 u | Zinc finger protein 395 |
| **Up-regulated genes** | | | | | |
| **WT** | **dKO** | **Ratio** | **p-value** | **Gene ID** | **Gene Name** |
| 0.998 | 3.385 | 5.23 | 0.0209 | NM_133662 $ | Immediate early response 3 |
| -4.165 | -1.928 | 4.71 | 0.0020 | Y16256 # | Basigin |
| -3.966 | -2.025 | 3.84 | 0.0054 | AW538932 & | RIKEN cDNA 2310016C08 gene |
| 0.293 | 1.699 | 2.65 | 0.0483 | NM_199029 # | Zinc finger protein 395 |
| 2.615 | 3.769 | 2.22 | 0.0085 | NM_053207 * | EGL nine homolog 1 (C. elegans) |
| -2.205 | -1.144 | 2.09 | 0.0476 | NM_011844 & | Monoglyceride lipase |
| -2.520 | -1.460 | 2.09 | 0.0167 | AV117762 $ | Moderately similar to NP_005557.1 lactate dehydrogenase A |
| 3.918 | 4.906 | 1.98 | 0.0278 | NM_013602 * | Metallothionein 1 |
| 1.514 | 2.444 | 1.91 | 0.0135 | NM_133232 # | 6-phosphofructo-2-kinase/fructose-2,6-biphosphatase 3 |
| 2.805 | 3.675 | 1.83 | 0.0261 | NM_007585 $ # | Annexin A2 |
| 2.867 | 3.615 | 1.68 | 0.0400 | NM_023168 # | Glutamate receptor, ionotropic, N-methyl D-aspartate-associated protein 1 |
| 1.385 | 2.106 | 1.65 | 0.0153 | NM_009396 * | Tumor necrosis factor, alpha-induced protein 2 |
| 3.025 | 3.721 | 1.62 | 0.0352 | NM_173866 * | Glutamic pyruvate transaminase (alanine aminotransferase) 2 |
| 6.253 | 6.946 | 1.62 | 0.0104 | NM_009768 # | Basigin |
| 0.000 | 0.672 | 1.59 | 0.0170 | NM_010393 # | Histocompatibility 2, D region locus 1 |
| 3.252 | 3.901 | 1.57 | 0.0148 | NM_010358 *# | Glutathione S-transferase, mu 1 |
| 1.652 | 2.274 | 1.54 | 0.0128 | NM_008183 *# | Glutathione S-transferase, mu 2 |
| 0.772 | 1.380 | 1.52 | 0.0157 | NM_010291 * | Gap junction protein, beta 5 |
| -0.713 | -0.115 | 1.51 | 0.0185 | AA989737 # | RIKEN cDNA 1700006J14 gene |
| 1.996 | 2.578 | 1.5 | 0.0017 | BE572994 $ | Laminin, alpha 3 |
| **Down-regulated genes** | | | | | |
| **WT** | **dKO** | **Ratio** | **p-value** | **Gene ID** | **Gene Name** |
| 1.235 | 0.269 | 1.95 | 0.0022 | NM_008765 # | Origin recognition complex, subunit 2-like (S. cerevisiae) |
| 0.930 | -0.033 | 1.95 | 0.0063 | NM_019769 # | RIKEN cDNA 1500003O03 gene |
| 2.469 | 1.533 | 1.91 | 0.0017 | NM_177474 # | DNA segment, Chr 19, Brigham & Womens Genetics 1357 expressed |
| 2.158 | 1.282 | 1.84 | 0.0004 | BE848537 & | Translocase of inner mitochondrial membrane 8 homolog a1 (yeast) |
| 1.252 | 0.410 | 1.79 | 0.0397 | NM_009772 & | Budding uninhibited by benzimidazoles 1 homolog (S. cerevisiae) |
| 1.692 | 0.861 | 1.78 | 0.0050 | NM_011450 & | trans-acting transcription factor 3 |
| 3.670 | 2.837 | 1.78 | 0.0012 | NM_008808 # | Platelet derived growth factor, alpha |
| 0.160 | -0.675 | 1.78 | 0.0009 | NM_007483 $ | Ras homolog gene family, member B |
| 4.856 | 4.039 | 1.76 | 0.0010 | NM_019796 # | Synaptotagmin binding, cytoplasmic RNA interacting protein |
| -1.794 | -2.604 | 1.75 | 0.0250 | NM_015779 * | Elastase 2, neutrophil |
| 2.850 | 2.042 | 1.75 | 0.0050 | NM_013814 * | Polypeptide N-acetylgalactosaminyltransferase 1 |
| 2.718 | 1.928 | 1.73 | 0.0377 | NM_016859 & | Bystin-like |
| -0.108 | -0.871 | 1.7 | 0.0176 | AK087461 #& | Cyclin-dependent kinase inhibitor 2C (p18, inhibits CDK4) |
| 3.351 | 2.589 | 1.7 | 0.0242 | NM_010849 &#* | Myelocytomatosis oncogene |
| 4.383 | 3.611 | 1.71 | 0.0011 | NM_053089 # | NMDA receptor-regulated gene 1 |
| 4.104 | 3.332 | 1.71 | 0.0488 | NM_008466 $ | Karyopherin (importin) alpha 3 |
| 3.015 | 2.272 | 1.67 | 0.0073 | NM_021463 $ | Phosphoribosyl pyrophosphate synthetase 1 |
| 3.510 | 2.782 | 1.66 | 0.0023 | NM_019553 # | DEAD (Asp-Glu-Ala-Asp) box polypeptide 21 |
| 4.987 | 4.268 | 1.65 | 0.0358 | NM_053120 # | heat shock protein 1 (chaperonin 10), pseudogene 2 (Hspe1-ps2), mRNA |
| 3.741 | 3.019 | 1.65 | 0.0030 | AK122322 # | Peroxisome proliferative activated receptor, gamma, coactivator-related 1 |
| 1.232 | 0.520 | 1.64 | 0.0042 | AK090391 # | RIKEN cDNA 2310061F22 gene |
| 5.194 | 4.506 | 1.61 | 0.0004 | NM_015751 # | ATP-binding cassette, sub-family E (OABP), member 1 |
| 2.003 | 1.338 | 1.58 | 0.0013 | NM_172722 # | RIKEN cDNA C330023M02 gene |
| 2.534 | 1.898 | 1.55 | 0.0135 | NM_145614 # | Dihydrolipoamide S-acetyltransferase |
| 4.295 | 3.675 | 1.54 | 0.0175 | BG793547 # | Cold shock domain protein A |
| 4.238 | 3.617 | 1.54 | 0.0030 | NM_145354 # | NOL1/NOP2/Sun domain family 2 |
| 2.606 | 1.994 | 1.53 | 0.0167 | NM_011992 * | Reticulocalbin 2 |
| 6.219 | 5.611 | 1.52 | 0.0030 | CF751402 # | Proliferating cell nuclear antigen |
| 1.140 | 0.542 | 1.51 | 0.0190 | NM_126165 # | Vacuolar protein sorting 4a (yeast) |
| 3.718 | 3.137 | 1.5 | 0.0029 | AK079406 # | Signal transducer and activator of transcription 3 |

d means down regulated genes, while u means up regulated one;

* Based on Valcourt et al.

# Based on Karlsson et al.

& Based on Yang et al.

$ Based on Chambers et al.

**Supplementary References:**

1. Valcourt U, Kowanetz M, Niimi H, Heldin CH, Moustakas A (2005) TGF-beta and the Smad signaling pathway support transcriptomic reprogramming during epithelial-mesenchymal cell transition. Molecular biology of the cell 16: 1987-2002.

2. Karlsson G, Liu Y, Larsson J, Goumans MJ, Lee JS, et al. (2005) Gene expression profiling demonstrates that TGF-beta1 signals exclusively through receptor complexes involving Alk5 and identifies targets of TGF-beta signaling. Physiological genomics 21: 396-403.

3. Yang YC, Piek E, Zavadil J, Liang D, Xie D, et al. (2003) Hierarchical model of gene regulation by transforming growth factor beta. Proceedings of the National Academy of Sciences of the United States of America 100: 10269-10274.

4. Chambers RC, Leoni P, Kaminski N, Laurent GJ, Heller RA (2003) Global expression profiling of fibroblast responses to transforming growth factor-beta1 reveals the induction of inhibitor of differentiation-1 and provides evidence of smooth muscle cell phenotypic switching. The American journal of pathology 162: 533-546.
