# Supplementary material for: Transcriptional Corepressors HIPK1 and HIPK2 Control Angiogenesis Via TGF-β–TAK1–Dependent Mechanism
Source: PLoS Biol. 2013 Apr 2;11(4):e1001527. doi: 10.1371/journal.pbio.1001527 (PMC3614511; doi:10.1371/journal.pbio.1001527)
Supplement: Table S2 — List of primer sequences (5′ to 3′) used in this study. (DOC) [file pbio.1001527.s009.doc]

| **Primers for qRT-PCR** | | |
| --- | --- | --- |
| **36B4** | Forward | GCGACCTGGAAGTCCAACTAC |
|  | Reverse | ATCTGCTGCATCTGCTTGG |
| **Pprc1** | Forward | CAGGAGAAGAAGCCCTTAGACC |
|  | Reverse | CTTTCGCCAAGAGTGAGACAG |
| **Cdkn2c** | Forward | CCTTGGGGGAACGAGTTGG |
|  | Reverse | AAATTGGGATTAGCACCTCTGAG |
| **Zfp395** | Forward | CCTGTCCCCACTCACCTCTA |
|  | Reverse | GTCCAGGCTGAAAGGCCAC |
| **Hmgb1** | Forward | GAGTACCGCCCAAAAATCAA |
|  | Reverse | CATAGGGCTGCTTGTCATCC |
| **Pcna** | Forward | TTTGAGGCACGCCTGATCC |
|  | Reverse | GGAGACGTGAGACGAGTCCAT |
| **Pprc1** | Forward | GCTTGGCTGTAGGAAACTCAG |
|  | Reverse | CAGTTCTGGGGCTTGTAACC |
| **Epo** | Forward | TAGCCTCACTTCACTGCTTCG |
|  | Reverse | GCTTGCAGAAAGTATCCACTGT |
| **Ier3** | Forward | GCTCTGGTCCCGAAATTTTCA |
|  | Reverse | AGATGATGGCGAACAGGAGAA |
| **Pai1** | Forward | TTCAGCCCTTGCTTGCCTC |
|  | Reverse | ACACTTTTACTCCGAAGTCGGT |
| **Alk1** | Forward | GGGCCTTTTGATGCTGTCG |
|  | Forward | TGGCAGAATGGTCTCTTGCAG |
| **Alk5** | Forward | TCCCAACTACAGGACCTTTTTCA |
|  | Reverse | GCAGTGGTAAACCTGATCCAGA |
| **Pecam** | Forward | CTGCCAGTCCGAAAATGGAAC |
|  | Reverse | CTTCATCCACCGGGGCTATC |
| **Nkx2.5** | Forward | ATAGATAGATAGACAGGCAG |
|  | Reverse | TACCCTTTACCAATTGCCAGC |
| **Ccne2** | Forward | ATGTCAAGACGCAGCCGTTTA |
|  | Forward | GCTGATTCCTCCAGACAGTACA |
| **Nkx2.1** | Forward | AGGACACCATGCGGAACAG |
|  | Reverse | CCATGCCGCTCATATTCATGC |
| **Gata1** | Forward | TGGGGACCTCAGAACCCTTG |
|  | Reverse | GGCTGCATTTGGGGAAGTG |
| **Fgf18** | Forward | CCTGCACTTGCCTGTGTTTAC |
|  | Reverse | TGCTTCCGACTCACATCATCT |
| **Slbp** | Forward | TGCAAGGTACAAAAGAAAACTGC |
|  | Forward | ACTTCCCGATGATGACTTCCTC |
| **Gata6** | Forward | TTGCTCCGGTAACAGCAGTG |
|  | Reverse | GTGGTCGCTTGTGTAGAAGGA |
| **Baf60c** | Forward | CCCGAGTCCCAGGCTTACA |
|  | Reverse | GCTTTCGCTTTTGCTTCATGG |
| **Nkx2.5** | Forward | GACAAAGCCGAGACGGATGG |
|  | Reverse | CTGTCGCTTGCACTTGTAGC |
| **Hdac7** | Forward | GGCAGGCTTACACCAGCAA |
|  | Forward | GGCTCAAGAGTTCTGTAGGGAA |
| **Vegfa** | Forward | AAAAACGAAAGCGCAAGAAA |
|  | Reverse | TTCTCCGCTCTGAACAAGG |
| **Gsc** | Forward | TACACGGGGACTCGCTCTAC |
|  | Reverse | GGTAGAAGGCGCCGTAGTC |
| **Ang2** | Forward | CCTCGACTACGACGACTCAGT |
|  | Reverse | TCTGCACCACATTCTGTTGGA |
| **Mmp10-1** | Forward | GAGCCACTAGCCATCCTGG |
|  | Forward | CTGAGCAAGATCCATGCTTGG |
| **Mmp10-4** | Forward | CCCTGGATTTTATGGAGATGTTC |
|  | Reverse | TGGGCTTGTGGAGAACCTGTAGAC |
| **Mmp1a-2** | Forward | TGTGTTTCACAACGGAGACC |
|  | Reverse | GCCCAAGTTGTAGTAGTTTTCCA |
| **Hipk1** | Forward | AGTTTAGCCCACTGCCACTC |
|  | Reverse | TCAGCTTCATCAGGGCTGT |
| **Hipk2** | Forward | AATTTGTGCCCGACCTGATC |
|  | Forward | ACTGAGTAGCCAGCGTGCTT |
| **Primers for ChIP assay** | | |
| **Mouse Mmp10** | Forward | CCCATGTGAGTCATGCTTAGTGA |
|  | Reverse | CCTGGTTTCCATATCTTCCCTTTA |
| **Human MMP10** | Forward | AACCTCTATGGTACAGC |
|  | Reverse | TCTCAGCTACTTGGAAG |
| **Primers for Gel shift assay** | | |
| **Mmp10-probe** | Forward | GTTCTTGTAGTCATTTGGATTAAAAATAGCACCCATGTGAGTCATGC |
|  | Forward | GCATGACTCACATGGGTGCTATTTTTAATCCAAATGACTACAAGAAC |
| **Mutant probe** | Reverse | GTTCTTGTAGTCATTTGGATTAGGGGTAGCACCCATGTGAGTCATGC |
|  | Forward | GCATGACTCACATGGGTGCTACCCCTAATCCAAATGACTACAAGAAC |
| **Primers for constructs** | | |
| **Vegfa-Luc** | Forward | CTGACTAGACCGGTACCTCTACTGTCTGGATGG |
|  | Reverse | GTCTACCATGGTCGCGACTGGTCCGATGCAAGATCC |
| **Vegfa-mMEF2** | Forward | CCCCCCCCCACAGGCAACATAGGGGTATTTTTGTTGCTACTTCATAAC |
|  | Forward | GTTATGAAGTAGCAACAAAAATACCCCTATGTTGCCTGTGGGGGGGGG |
| **Vegfa-mSBE** | Forward | GCAAATATTAATTACCCGCGTAGTCCCTTTTCCTCTTTGTAAACAC |
|  | Reverse | GTGTTTACAAAGAGGAAAAGGGACTACGCGGGTAATTAATATTTGC |
| **Mmp10-mSBE** | Forward | CATTTTGCAAATATTAATTACCCGCGTAGTCCCTTTTCCTCTTTGTAAAC |
|  | Reverse | GTTTACAAAGAGGAAAAGGGACTACGCGGGTAATTAATATTTGCAAAATG |
| **Hipk2-S359A** | Forward | CATGTGTCCAAAGCTGTCTGCGCTACGTACTTGCAATCCAGATACTACC |
|  | Reverse | GGTAGTATCTGGATTGCAAGTACGTAGCGCAGACAGCTTTGGACACATG |
| **Hipk2-T360A** | Forward | GTGTCCAAAGCTGTCTGCTCTGCGTACTTGCAATCCAGATACTACCGGG |
|  | Forward | CCCGGTAGTATCTGGATTGCAAGTACGTAGCGCAGACAGCTTTGGACAC |
| **Hipk2-Y361F** | Forward | CCAAAGCTGTCTGCTCTACGTTCTTGCAATCCAGATACTACCGGGCCC |
|  | Reverse | GGGCCCGGTAGTATCTGGATTGCAAGTACGTAGCGCAGACAGCTTTGG |
